# Association Between C‐Reactive Protein–Triglyceride Glucose Index and All‐Cause and Cardiovascular Mortality Across Cardiovascular–Kidney–Metabolic Syndrome Stages 0–4

**DOI:** 10.1155/ije/6690501

**Published:** 2026-07-30

**Authors:** Siqi Yi, Weida Qiu, Yanchen Zhu, Shiping Wu, He Zheng, Qiujin Huang, Yingqing Feng, Ying Wu

**Affiliations:** ^1^ Guangdong Provincial Key Laboratory of Coronary Heart Disease Prevention, Guangdong Cardiovascular Institute, Guangdong Provincial People’s Hospital (Guangdong Academy of Medical Sciences), Southern Medical University, Guangzhou 510080, China, fimmu.com; ^2^ School of Medicine, South China University of Technology, Guangzhou, China, scut.edu.cn

**Keywords:** cardiovascular-kidney-metabolic, C-reactive protein-triglyceride glucose index, inflammation, insulin resistance, mortality, national health and nutrition examination survey

## Abstract

**Objective:**

Advanced cardiovascular–kidney–metabolic (CKM) syndrome corresponds to an elevated risk of mortality. The C‐reactive protein–triglyceride glucose index (CTI) reflects both insulin resistance and inflammation, processes that are crucial to CKM progression. However, it remains uncertain whether CTI is linked to mortality risk and how this relationship may vary across different CKM progression stages.

**Methods:**

A total of 8314 participants were drawn from American adults. Participants were then grouped by advanced CKM stages. CTI was calculated as 0.412 × Ln (CRP [mg/L]) + Ln (TG [mg/dl] × FBG [mg/dl])/2. The endpoints focused on all‐cause and cardiovascular mortality. Associations of CTI with advanced CKM stages and mortality were examined using logistic and Cox regression models, respectively.

**Results:**

The highest CTI quartile (Q4) exhibited a markedly greater prevalence of advanced CKM stages relative to Q1 (*p* < 0.001). During a median follow‐up of 152 months, 1606 participants died, including 475 cardiovascular deaths. Each SD increase in CTI was associated with a 35% higher risk of all‐cause mortality (95% CI: 1.24–1.48) and a 40% higher risk of cardiovascular mortality (95% CI: 1.20–1.62). Similarly, compared with Q1, participants in Q4 of CTI had higher risks of all‐cause mortality (HR = 1.82, 95% CI: 1.46–2.27) and cardiovascular mortality (HR = 2.29, 95% CI: 1.56–3.34), with homogeneous associations across different CKM stages (interaction *p* > 0.05).

**Conclusion:**

Higher CTI levels were accompanied by a greater prevalence of advanced CKM stages and were associated with increased risks of all‐cause and cardiovascular mortality across CKM stages. CTI may provide complementary information for mortality‐risk assessment although further validation is needed.

## 1. Introduction

Cardiovascular–kidney–metabolic (CKM) syndrome is characterized as a systemic disorder arising from the interplay among metabolic risk factors, chronic kidney disease (CKD), and cardiovascular diseases (CVDs) [1]. An expanding body of evidence has reinforced the notion of CKM syndrome, revealing the complex and close interplay connecting metabolic disorders, CKD, and CVD [2–5]. This interplay exerts a profound impact on mortality, exceeding the simple sum of its individual components [6]. The 2021 Global Burden of Disease research reported a global CVD incidence of 523 million cases, approximately twice as many as in 1990, confirming CVD as the most prominent cause of mortality worldwide [7]. From 2015 to 2020, research indicated that CKM syndrome impacted more than a quarter of U.S. adults and emerged as a significant cause of death during this period [8, 9]. Therefore, it is imperative to manage all facets of CKM syndrome as an integrated whole to reduce mortality. In addition, much of the clinical burden associated with CKM syndrome remains disproportionately attributable to CVD [10]. To help with prevention and management, the American Heart Association (AHA) proposed a staging framework classifying CKM syndrome from Stages 0–4 and prioritized cardiovascular event prevention for individuals in Stages 0–3.

Identified as a surrogate biomarker indicator of insulin resistance (IR) [11], the triglyceride glucose (TyG) index shows strong correlations with diabetes, metabolic syndrome (MeTS), CKD, and CVD [12–15]. Furthermore, inflammation, as a shared pathophysiological basis of many chronic diseases, drives atherosclerosis, kidney injury, and metabolic disturbances associated with IR through proinflammatory cytokines [16]. Various biomarkers reflecting systemic inflammation have been proposed, among which C‐reactive protein (CRP) remains a widely recognized example [17]. The C‐reactive protein–triglyceride glucose index (CTI), which combines TyG and CRP, is a composite indicator that may reflect both IR and systemic inflammation [18]. The foregoing evidence underscores CTI’s clinical potential. Furthermore, CTI has demonstrated predictive value for diabetes risk, coronary heart disease prevalence, cancer mortality, and stroke risk [19–22]. However, it remains uncertain whether CTI is linked to mortality risk. Additionally, considering the interactive relationships between metabolic disorders, inflammation, and disease progression, it remains to be elucidated whether CTI’s link to mortality varies across different CKM syndrome stages.

Accordingly, we utilized National Health and Nutrition Examination Survey (NHANES) data to investigate how CTI relates to the prevalence of advanced CKM Stages (3‐4). We examined how CTI is associated with mortality risk across CKM syndrome Stages 0–4. Elucidating these relationships is crucial for early prevention and mortality risk identification.

## 2. Materials and Methods

### 2.1. Study Design and Population

NHANES is a nationwide study that adopts a complex, multistage probability sampling approach to evaluate the health and nutrition of noninstitutionalized American adults and children. Data are collected via interviews, comprehensive physical examinations, and laboratory assessments. The protocol received approval from the National Center for Health Statistics (NCHS) Ethics Review Board, and all participants provided informed consent.

This study incorporated six consecutive NHANES cycles spanning 1999–2010, initially including 62,160 participants. Those younger than 20 years and those who were pregnant were first excluded. Next, we excluded participants whose cardiovascular risk—employed in CKM staging—was not calculable via Predicting risk of CVD EVENTs (PREVENT) equations. We also excluded participants missing CRP, triglyceride (TG), or fasting blood glucose (FBG) required to calculate the CTI, those sample weights (WTSAF2YR) = 0, and those lacking follow‐up data. Ultimately, 8314 eligible individuals were retained in the final analytic cohort (Figure S1). To assess potential selection bias due to exclusion of participants with missing CTI‐related variables, we further compared baseline characteristics between included participants and those excluded because of missing CRP, FBG, or TG. The two groups were broadly similar across most baseline characteristics although some differences were observed in several clinical measures (Table S1).

### 2.2. Calculation of CTI

Formula for calculating CTI is as follows [20]: CTI = 0.412 × Ln (CRP [mg/L]) + Ln (TG [mg/dl] × FBG [mg/dl])/2.

### 2.3. Definition of Endpoint Event

Study endpoints comprised all‐cause and cardiovascular mortality, identified in accordance with ICD‐10 coding. All‐cause mortality encompassed deaths attributable to any cause, while cardiovascular mortality (054–068) included fatalities due to cardiac conditions and cerebrovascular diseases as classified by ICD‐10 codes. Mortality status was determined through probabilistic linkage of NHANES participant data with death certificate records from the National Death Index, as compiled by the NCHS, with follow‐up updated through 31 December 2019.

### 2.4. Definition of CKM Syndrome Stage

In accordance with the AHA statement, CKM syndrome was categorized into five distinct stages (Table 1). Specifically, Stage 0 indicates no CKM risk factors; Stage 1 is defined by dysfunctional or excessive obesity; Stage 2 includes those exhibiting metabolic abnormalities such as hypertension, MeTS, diabetes or hypertriglyceridemia (TG ≥ 135 mg/dL), or moderate‐to‐high risk CKD; Stage 3 corresponds to subclinical CVD; and Stage 4 corresponds to clinically diagnosed CVD. In this study, participants classified as Stage 3 or Stage 4 are aggregated to define the advanced CKM stages [1, 16, 23, 24]. More detailed definitions of diseases and the specific criteria used for CKM staging are provided in Tables S2 and S3.

**TABLE 1:** Simplified operational definitions of CKM syndrome stages in NHANES.

CKM stages	Definition
CKM Stage 0	No CKM risk factors
CKM Stage 1	Obesity or prediabetes
CKM Stage 2	Diabetes, hypertension, hypertriglyceridemia, metabolic syndrome, or moderate‐to‐high‐risk CKD
CKM Stage 3	10‐year PREVENT CVD risk ≥ 20% or very‐high‐risk CKD
CKM Stage 4	Established cardiovascular disease

*Note:* PREVENT, Predicting Risk of Cardiovascular Disease Events.

Abbreviations: CKD, chronic kidney disease; CKM, cardiovascular–kidney–metabolic; CVD, cardiovascular disease; NHANES, National Health and Nutrition Examination Survey.

Subclinical CVD refers to either a predicted 10‐year cardiovascular risk of at least 20% or very‐high‐risk CKD. We estimated the 10‐year cardiovascular risk through implementation of PREVENT equations developed by the AHA [25]; the specific formulas are provided in Table S4. CKD risk is classified based on measures of renal function and damage, specifically estimated glomerular filtration rate (eGFR) and urine albumin‐to‐creatinine ratio (UACR) [26]. eGFR is calculated with the CKD‐EPI Creatinine Age‐Sex Equation (2021) [27].

### 2.5. Data Collection

We collected the following data for this study: (1) Demographic characteristics, including age, poverty income ratio (PIR), gender, race, marital, and education. (2) Body measurements, such as systolic blood pressure (SBP), diastolic blood pressure, body mass index (BMI), and waist circumference (WC). (3) Lifestyle data, including smoking and drinking. (4) Medical history and medication use, covering liver disease, cancer, diabetes, MeTS, hypertension, antihypertensive drugs, antihyperglycemic drugs, and statin use. (5) Laboratory examination, comprising low‐density lipoprotein cholesterol (LDL), high‐density lipoprotein cholesterol (HDL), total cholesterol (TC), FBG, TG, hemoglobin A1c (HbA1c), CRP, uric acid (UA), urine creatinine, urine albumin, and serum creatinine.

### 2.6. Missing Data Handling

Table S5 details the proportion of missing variables included in the analysis. To handle missing values and reduce potential bias, we used the random forest method implemented with the mice package in R, generating five imputed datasets with 10 iterations (*m* = 5, maxit = 10, method = “rf”). Imputation was performed using the analytic dataset, which included the variables used in the main analyses. The variables with missing values and, therefore, imputed included PIR, drinking, LDL, marital, WC, UACR, antihypertensive drugs, liver disease, HbA1c, education, smoking, cancer, and antihyperglycemic drugs.

### 2.7. Statistical Analysis

To derive nationally representative and reliable estimates for the noninstitutionalized American population, the analyses incorporated fasting subsample weights (WTSAF2YR and WTSAF4YR) alongside design adjustments for clustering and stratification. We pooled data from six survey cycles (1999–2010) and constructed a 12‐year fasting subsample weight following NHANES analytic guidelines, using (2/6) × WTSAF4YR for 1999–2002 and (1/6) × WTSAF2YR for 2003–2010. Based on CTI quartiles (Q1–Q4), participants were categorized into four distinct groups. Normality of continuous variables was assessed via the Kolmogorov–Smirnov test, revealing that all failed to meet a normal distribution (Table S6). Accordingly, these variables were reported as medians with interquartile ranges, and group comparisons were performed using the Kruskal–Wallis test. For categorical variables, data were presented as frequencies and percentages, with intergroup differences evaluated through chi‐square analysis.

At baseline, we conducted a cross‐sectional analysis using logistic regression to assess the association between CTI and advanced CKM stages. For mortality outcomes, the study was conducted as a prospective cohort analysis using baseline CTI to examine its association with subsequent events. Survival probabilities across CTI quartiles were compared using Kaplan–Meier curves. Schoenfeld residuals were used to assess whether the proportional‐hazards assumption of the Cox model was satisfied. Cox regression models were then applied to examine the link between CTI and mortality among the overall participants, as well as within two groups stratified by advanced CKM stages. CTI was modeled both as a continuous variable (per SD increase) and as a categorical variable divided into quartiles (Q1–Q4), with Q1 serving as the reference group. The P for trend was calculated by including the quartiles as an ordinal variable (1–4) to assess the dose‐response relationship. Covariates were selected a priori based on previous literature and clinical relevance, particularly their potential roles as confounders in the associations of CTI with CKM stage and mortality outcomes [28–30]. Four statistical models with sequential adjustments were developed: Model 1 remained unadjusted; Model 2 additionally controlled for age, gender, and race; Model 3 further incorporated marital, educational attainment, smoking, and alcohol consumption; and Model 4 was further expanded to adjust for PIR, BMI, SBP, HDL, LDL, HbA1c, UA, eGFR, UACR, statin use, antihypertensive drugs, antihyperglycemic drugs, liver disease, and cancer. Models 1–3 were used to illustrate the sequential impact of covariate adjustment, while Model 4 was specified as the primary fully adjusted model for inference. To assess potential multicollinearity within each model, variance inflation factors (VIFs) were calculated. A VIF value below 5 was considered to indicate the absence of notable multicollinearity. All VIF values were below this threshold (Table S7). Time‐dependent ROC analyses were performed using the timeROC package in R to compare the exploratory marker‐level discrimination of CTI, TyG, and CRP for all‐cause and cardiovascular mortality at 5 and 10 years. Pairwise AUC comparisons between CTI and TyG or CRP were performed using the influence‐function‐based test implemented in the timeROC package with iid = TRUE, and adjusted *p* values were reported to account for multiple time‐point comparisons. These exploratory ROC analyses did not incorporate the full NHANES complex survey design, whereas the primary regression analyses incorporated fasting subsample weights, clustering, and stratification. We further evaluated the incremental predictive value of CTI beyond the reference clinical covariate model using Harrell’s C statistic, continuous net reclassification improvement (NRI), and integrated discrimination improvement (IDI). NRI and IDI were calculated for 10‐year mortality risk. Additionally, subgroup and interaction analyses examined how CTI related to mortality across different subpopulations. Analyses were stratified by demographic and clinical factors, including age, gender, race, smoking and drinking behavior, and the presence of diabetes or hypertension.

Several sensitivity analyses were conducted to enhance confidence in the stability of results. First, to mitigate possible reverse causality, individuals who died during the initial 2 years of follow‐up were excluded. Second, considering the substantial impact of cancer on mortality, individuals with a self‐reported history of cancer at baseline were omitted. Third, a complete‐case analysis was performed by removing subjects with missing covariate data. Fourth, to avoid obscuring important differences caused by combining subclinical and clinical CVD within advanced CKM Stages (3–4), CKM was further reclassified into nonadvanced CKM stages, CKM Stage 3, and CKM Stage 4. Fifth, to examine whether the observed pattern was limited to the binary definition of advanced CKM stages, we conducted an additional sensitivity analysis by reclassifying CKM into Stages 0–4 and examining the association using ordinal logistic regression models. Sixth, to address potential overadjustment, we additionally performed a parsimonious adjustment analysis excluding cardiometabolic, renal, and medication‐related variables that could plausibly lie on the pathway between CTI and mortality. This model adjusted for age, gender, race, marital status, education, PIR, smoking, drinking, liver disease, and cancer.

Statistical analyses were conducted in R (v4.4.2), with two‐tailed *p* < 0.05 deemed significant.

## 3. Results

### 3.1. Baseline Characteristics of Participants Stratified by Advanced CKM Stages

Table 2 presents participants’ baseline characteristics stratified by advanced CKM stages. This study enrolled 8314 participants, of whom 47.9% were female, with a median age of 49.0 years (IQR: 35.0–64.0), and 51.1% Non‐Hispanic White. Participants classified as advanced CKM stages were generally older, predominantly male, had lower educational attainment and wealth, and showed reduced prevalence of smoking and alcohol consumption alongside worse metabolic profiles. Additionally, their BMI, WC, SBP, FBG, HbA1c, TG, CRP, UA, and UACR were elevated, whereas TC, HDL, and eGFR were comparatively lower. The participants’ baseline characteristics stratified by quartile of CTI are presented in Table S8.

**TABLE 2:** Baseline characteristics of participants classified by advanced CKM stages.

Variables	Overall	Nonadvanced CKM stages	Advanced CKM stages	*p* value
Total	8314	6943	1371	
Age, years	49.00 (35.00, 64.00)	45.00 (33.00, 59.00)	73.00 (64.00, 80.00)	< 0.001
Gender, female, *n* (%)	3985 (47.9)	3523 (50.7)	462 (33.7)	< 0.001
Race, *n* (%)				< 0.001
Mexican American	1750 (21.0)	1547 (22.3)	203 (14.8)	
Other Hispanic	649 (7.8)	570 (8.2)	79 (5.8)	
Non‐Hispanic White	4249 (51.1)	3416 (49.2)	833 (60.8)	
Non‐Hispanic Black	1359 (16.3)	1137 (16.4)	222 (16.2)	
Other race	307 (3.7)	273 (3.9)	34 (2.5)	
PIR	2.34 (1.24, 4.32)	2.45 (1.25, 4.44)	1.96 (1.17, 3.50)	< 0.001
Marital, *n* (%)	5345 (64.3)	4472 (64.4)	873 (63.7)	0.626
Education, *n* (%)				< 0.001
Less than high school	2419 (29.1)	1872 (27.0)	547 (39.9)	
High school or equivalent	1969 (23.7)	1646 (23.7)	323 (23.6)	
College or above	3926 (47.2)	3425 (49.3)	501 (36.5)	
Smoking, *n* (%)				< 0.001
Never	4324 (52.0)	3782 (54.5)	542 (39.5)	
Former	2218 (26.7)	1623 (23.4)	595 (43.4)	
Current	1772 (21.3)	1538 (22.2)	234 (17.1)	
Drinking, *n* (%)	6082 (73.2)	5140 (74.0)	942 (68.7)	< 0.001
BMI, kg/m^2^	27.45 (24.33, 31.12)	27.33 (24.21, 30.98)	28.15 (25.01, 31.57)	< 0.001
Waist circumference, cm	96.50 (87.60, 105.90)	95.40 (86.50, 104.60)	102.40 (94.75, 110.50)	< 0.001
SBP, mmHg	120.67 (110.67, 133.33)	118.67 (110.00, 130.00)	134.00 (120.00, 150.67)	< 0.001
DBP, mmHg	70.00 (63.33, 77.33)	70.67 (64.00, 77.33)	68.00 (58.67, 76.00)	< 0.001
FBG, mg/dL	98.80 (92.00, 107.60)	97.10 (91.00, 105.00)	108.00 (98.00, 128.00)	< 0.001
HbA1c, %	5.40 (5.20, 5.70)	5.40 (5.10, 5.70)	5.80 (5.40, 6.35)	< 0.001
TG, mg/dL	107.00 (74.00, 158.00)	104.00 (72.00, 152.00)	125.00 (89.50, 180.00)	< 0.001
TC, mg/dL	196.00 (172.00, 224.00)	198.00 (174.00, 225.00)	188.00 (164.00, 216.00)	< 0.001
HDL, mg/dL	51.00 (42.00, 62.00)	52.00 (42.00, 63.00)	47.00 (40.00, 57.00)	< 0.001
LDL, mg/dL	118.00 (96.00, 142.00)	120.00 (98.00, 143.00)	110.00 (89.00, 134.00)	< 0.001
CRP, mg/dL	0.19 (0.08, 0.42)	0.18 (0.08, 0.40)	0.25 (0.12, 0.54)	< 0.001
UA, mg/dL	5.40 (4.50, 6.40)	5.30 (4.40, 6.20)	5.90 (5.00, 7.00)	< 0.001
UACR, mg/g	6.40 (4.10, 12.10)	5.90 (4.00, 10.40)	10.70 (5.80, 30.55)	< 0.001
eGFR, mL/min/1.73 m^2^	98.10 (82.60, 112.00)	101.40 (88.20, 114.20)	74.70 (57.75, 92.30)	< 0.001
Antihypertensive drugs, *n* (%)	2206 (26.5)	1304 (18.8)	902 (65.8)	< 0.001
Antihyperglycemic drugs, *n* (%)	674 (8.1)	345 (5.0)	329 (24.0)	< 0.001
Statin use, *n* (%)	1537 (18.5)	975 (14.0)	562 (41.0)	< 0.001
Hypertension, *n* (%)	4202 (50.5)	3044 (43.8)	1158 (84.5)	< 0.001
Diabetes, *n* (%)	1122 (13.5)	597 (8.6)	525 (38.3)	< 0.001
MeTS, *n* (%)	3089 (37.2)	2247 (32.4)	842 (61.4)	< 0.001
Liver disease, *n* (%)	288 (3.5)	216 (3.1)	72 (5.3)	< 0.001
Cancer, *n* (%)	744 (8.9)	452 (6.5)	292 (21.3)	< 0.001
CKD, *n* (%)				< 0.001
Low risk	7090 (85.3)	6313 (90.9)	777 (56.7)	
Moderate to high risk	1120 (13.5)	630 (9.1)	490 (35.7)	
Very high risk	104 (1.3)	0 (0.0)	104 (7.6)	

*Note:* Normality was assessed using the Kolmogorov–Smirnov test, and all continuous variables were nonnormally distributed, thus described as median (interquartile range); categorical variables were expressed as frequency (percentage).

Abbreviations: BMI, body mass index; CKD, chronic kidney disease; CKM, cardiovascular–kidney–metabolic; CRP, C‐reactive protein; CTI, C‐reactive protein–triglyceride glucose index; DBP, diastolic blood pressure; eGFR, estimated glomerular filtration rate; FBG, fasting blood glucose; HbA1c, hemoglobin A1c; HDL, high density lipoprotein cholesterol; LDL, low density lipoprotein cholesterol; MeTS, metabolic syndrome; PIR, ratio of family income to poverty; SBP, systolic blood pressure; TC, total cholesterol; TG, triglyceride; UA, uric acid; UACR, urine albumin‐to‐creatinine ratio.

### 3.2. CKM Syndrome Stage Prevalence Across CTI Quartiles

The prevalence of CKM Stages 0–4 was 10.3%, 20.0%, 53.2%, 7%, and 9.5% (Table S8). Importantly, the distribution of CKM stages varied markedly across CTI quartiles. Compared with participants with lower CTI, those in higher CTI groups exhibited substantially fewer cases in CKM Stages 0–1 and more cases clustered in Stages 2–4 (*p* < 0.001). Notably, the prevalence of advanced CKM stages rose steadily as the CTI quartiles increased (*p* < 0.001).

### 3.3. Association Between CTI and Advanced CKM Stages

In Table 3, CTI remained associated with higher odds of advanced CKM stages after adjusting for age, gender, race, PIR, drinking, smoking, marital, education, SBP, BMI, HDL, LDL, HbA1c, UA, eGFR, UACR, statin use, antihypertensive drugs, antihyperglycemic drugs, liver disease, and cancer. After controlling for all covariates, a one standard deviation increase in CTI was associated with 23% higher odds of advanced CKM stages (OR = 1.23, 95% CI: 1.06–1.42). Quartile‐based analysis similarly indicated that participants classified in Q4 exhibited a significantly higher prevalence of advanced CKM stages compared to the Q1 group (*p* < 0.001). Analysis of RCS (Figure S2) revealed an approximately linear association between CTI and odds of advanced CKM stages.

**TABLE 3:** Association between CTI and advanced cardiovascular–kidney–metabolic stages.

	Model 1 OR (95% CI)	*p* value	Model 2 OR (95% CI)	*p* value	Model 3 OR (95% CI)	*p* value	Model 4 OR (95% CI)	*p* value
Per SD increase	1.79 (1.67, 1.91)	< 0.001	1.69 (1.53, 1.86)	< 0.001	1.59 (1.44, 1.76)	< 0.001	1.23 (1.06, 1.42)	0.007
Q1	Ref		Ref		Ref		Ref	
Q2	2.69 (2.11, 3.43)	< 0.001	1.93 (1.42, 2.62)	< 0.001	1.81 (1.33, 2.46)	< 0.001	1.47 (1.07, 2.03)	0.019
Q3	3.11 (2.50, 3.87)	< 0.001	1.85 (1.41, 2.43)	< 0.001	1.67 (1.26, 2.20)	< 0.001	1.19 (0.88, 1.62)	0.254
Q4	5.43 (4.40, 6.70)	< 0.001	3.78 (2.91, 4.91)	< 0.001	3.24 (2.47, 4.25)	< 0.001	1.89 (1.32, 2.70)	< 0.001
*P* for trend		< 0.001		< 0.001		< 0.001		0.005

*Note:* SD of CTI = 0.66. Model 1: unadjusted; Model 2: adjusted for age, gender, and race; Model 3: adjusted for age, gender, race, marital, education, smoking, and drinking; Model 4: adjusted for age, gender, race, marital, education, smoking, drinking, PIR, BMI, SBP, HDL, LDL, HbA1c, UA, eGFR, UACR, statin use, antihypertensive drugs, antihyperglycemic drugs, liver disease, and cancer.

Abbreviations: BMI, body mass index; CTI, C‐reactive protein–triglyceride glucose index; CI, confidence interval; eGFR, estimated glomerular filtration rate; HbA1c, hemoglobin A1c; HDL, high‐density lipoprotein cholesterol; LDL, low‐density lipoprotein cholesterol; OR, odds ratio; PIR, ratio of family income to poverty; SBP, systolic blood pressure; UA, uric acid; UACR, urine albumin‐to‐creatinine ratio.

### 3.4. Association Between CTI and All‐Cause and Cardiovascular Mortality Stratified by Advanced CKM Stages

During a median follow‐up of 152 months, 1606 participants died, including 475 cardiovascular deaths. Schoenfeld residual tests did not indicate significant violations of the proportional hazards assumption in the primary Cox models, with global *p* values > 0.05 for both all‐cause and cardiovascular mortality models. Kaplan–Meier curves (Figure S3) revealed marked variation in all‐cause and cardiovascular mortality among CTI quartiles, with the lowest rates in the bottom quartile. Among the overall participants (Table 4), each SD increase in CTI was associated with a 35% higher risk of all‐cause mortality (95% CI: 1.24–1.48) and a 40% higher risk of cardiovascular mortality (95% CI: 1.20–1.62). Similarly, compared with Q1, participants in Q4 of CTI had higher risks of all‐cause mortality (HR = 1.82, 95% CI: 1.46–2.27) and cardiovascular mortality (HR = 2.29, 95% CI: 1.56–3.34), with homogeneous associations across different CKM stages (*p* for interaction > 0.05).

**TABLE 4:** Association between CTI and all‐cause and cardiovascular mortality stratified by advanced CKM stages.

	All participants			Nonadvanced CKM stages			Advanced CKM stages			*p*‐interaction
	Events/total (%)	HR and 95% CI	*p* value	Events/total (%)	HR and 95% CI	*p* value	Events/total (%)	HR and 95% CI	*p* value	
All‐cause mortality										
Per SD increase	1606/8314 (19.3)	1.35 (1.24, 1.48)	< 0.001	799/6943 (11.5)	1.35 (1.19, 1.54)	< 0.001	807/1371 (58.8)	1.37 (1.22, 1.54)	< 0.001	0.172
Q1	194/2079 (9.3)	Ref	—	110/1929 (5.7)	Ref	—	84/150 (56.0)	Ref	—	0.429
Q2	364/2078 (17.5)	1.24 (1.01, 1.51)	0.035	183/1747 (10.4)	1.31 (0.99, 1.73)	0.062	181/331 (54.6)	1.13 (0.86, 1.48)	0.378	
Q3	466/2078 (22.4)	1.35 (1.09, 1.67)	0.006	246/1708 (14.4)	1.36 (1.02, 1.82)	0.038	220/370 (59.4)	1.37 (1.02, 1.83)	0.039	
Q4	582/2079 (27.9)	1.82 (1.46, 2.27)	< 0.001	260/1559 (16.6)	1.84 (1.32, 2.56)	< 0.001	322/520 (61.9)	1.76 (1.32, 2.35)	< 0.001	
*p* for trend	—	—	< 0.001	—	—	< 0.001	—	—	< 0.001	—
CVD mortality										
Per SD increase	475/8314 (5.7)	1.40 (1.20, 1.62)	< 0.001	204/6943 (2.9)	1.48 (1.20, 1.84)	< 0.001	271/1371 (19.7)	1.31 (1.07, 1.60)	0.010	0.282
Q1	53/2079 (2.5)	Ref	—	25/1929 (1.2)	Ref	—	28/150 (18.6)	Ref	—	0.454
Q2	114/2078 (5.4)	1.63 (1.11, 2.39)	0.014	44/1747 (2.5)	1.85 (1.08, 3.15)	0.024	70/331 (21.1)	1.25 (0.74, 2.10)	0.406	
Q3	133/2078 (6.4)	1.52 (1.01, 2.30)	0.045	69/1708 (4.0)	1.96 (1.08, 3.53)	0.026	64/370 (17.2)	1.11 (0.66, 1.88)	0.695	
Q4	175/2079 (8.4)	2.29 (1.56, 3.34)	< 0.001	66/1559 (4.2)	2.75 (1.57, 4.83)	< 0.001	109/520 (20.9)	1.67 (1.00, 2.81)	0.052	
*p* for trend	—	—	0.001	—	—	0.001	—	—	0.063	—

*Note:* Full adjustment for age, gender, race, marital, education, smoking, drinking, PIR, BMI, SBP, HDL, LDL, HbA1c, UA, eGFR, UACR, statin use, antihypertensive drugs, antihyperglycemic drugs, liver disease, and cancer. HRs for quartiles (Q2–Q4) were estimated by modeling CTI quartiles as categorical variables, using Q1 as the reference group. The P for trend was calculated by treating CTI quartiles as an ordinal variable (coded 1–4) in the fully adjusted Cox model. The denominator for each CKM subgroup remained unchanged across both the all‐cause and cardiovascular mortality analyses; cardiovascular deaths represent a subset of all‐cause deaths within the same analytic sample.

Abbreviations: BMI, body mass index; CI, confidence interval; CKM, cardiovascular–kidney–metabolic; CTI, C‐reactive protein–triglyceride glucose index; eGFR, estimated glomerular filtration rate; HbA1c, hemoglobin A1c; HDL, high‐density lipoprotein cholesterol; HR, hazard ratio; LDL, low‐density lipoprotein cholesterol; PIR, ratio of family income to poverty; SBP, systolic blood pressure; UA, uric acid; UACR, urine albumin‐to‐creatinine ratio.

**FIGURE 1:**
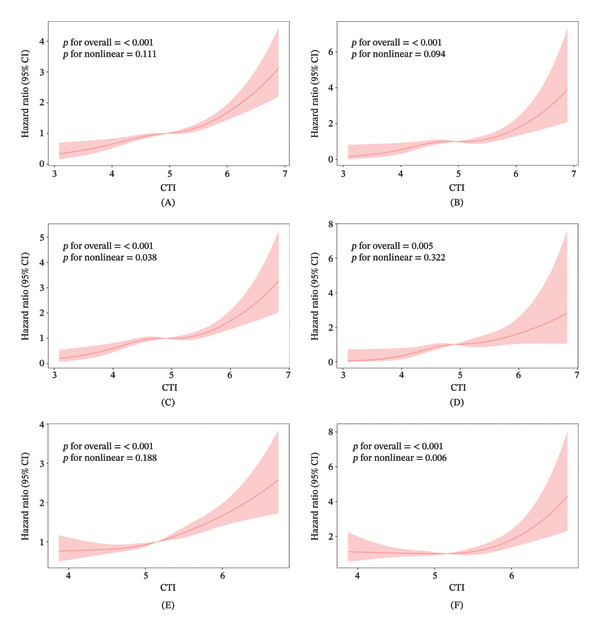
Restricted cubic splines illustrate the relationship between CTI and mortality in adults with different CKM stages. Full adjustment. (A) CTI with all‐cause mortality in CKM Stages 0–4; (B) CTI with cardiovascular mortality in CKM Stages 0–4; (C) CTI with all‐cause mortality in nonadvanced stages; (D) CTI with cardiovascular mortality in nonadvanced stages; (E) CTI with all‐cause mortality in advanced stages; and (F) CTI with cardiovascular mortality in advanced stages. CTI, C‐reactive protein–triglyceride glucose index; CKM, cardiovascular–kidney–metabolic.

RCS regression was conducted to explore whether CTI showed linear or nonlinear associations with mortality. The fully adjusted RCS models largely showed linear associations of CTI with all‐cause (Figure 1A) and cardiovascular mortality (Figure 1B). Interestingly, among participants at nonadvanced CKM stages, CTI displayed a curvilinear association with all‐cause mortality (Figure 1C), whereas its association with cardiovascular mortality remained largely linear (Figure 1D). Among participants at advanced CKM stages, the association with all‐cause mortality was largely linear (Figure 1E), while a nonlinear association emerged regarding cardiovascular mortality (Figure 1F).

In the time‐dependent ROC analyses (Figure S4), CTI generally showed higher AUCs than TyG and CRP across outcomes and time points. For all‐cause mortality, the AUCs of CTI were 0.640 at 5 years and 0.616 at 10 years, compared with 0.625 and 0.592 for CRP and 0.586 and 0.590 for TyG, respectively. Compared with TyG, CTI showed significantly higher AUCs for all‐cause mortality at both 5 and 10 years; compared with CRP, CTI showed a significantly higher AUC at 10 years, whereas the difference at 5 years was not statistically significant after adjustment for multiple comparisons. For cardiovascular mortality, CTI also showed higher AUCs overall. Compared with CRP, CTI showed significantly higher AUCs at both 5 and 10 years after adjustment for multiple comparisons, whereas the differences between CTI and TyG were not statistically significant at either time point. In the incremental predictive performance analysis, adding CTI to the reference clinical covariate model resulted in small improvements in discrimination and reclassification (Table S9). The C statistic increased from 0.865 to 0.867 for all‐cause mortality and from 0.884 to 0.887 for cardiovascular mortality, with absolute improvements of 0.2% for both outcomes. The corresponding NRI and IDI were 0.175 and 0.004 for all‐cause mortality and 0.280 and 0.004 for cardiovascular mortality, respectively.

### 3.5. Subgroup and Sensitivity Analysis

**FIGURE 2:**
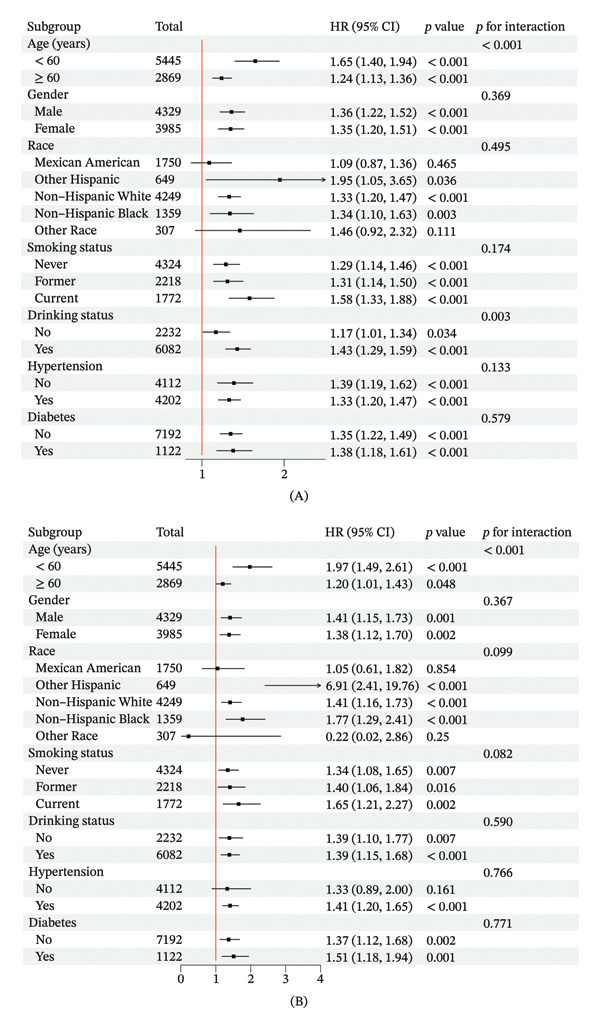
Subgroup and interaction analyses of the association between C‐reactive protein–triglyceride glucose index and all‐cause (A) and cardiovascular mortality (B). Full adjustment.

Subgroup analyses stratified by age, gender, race, alcohol and smoking consumption, diabetes, and hypertension were performed to explore how CTI relates to mortality within different demographic and clinical contexts. Figure 2 illustrates that, across all subgroups, higher CTI levels were associated with elevated risks of both all‐cause and cardiovascular mortality. Importantly, among participants younger than 60 years, CTI showed a notably stronger association with both all‐cause (interaction *p* < 0.001) and cardiovascular mortality (interaction *p* < 0.001). Additionally, its association with all‐cause mortality appeared more pronounced in those who reported alcohol consumption (interaction *p* = 0.003). No significant effect modifications of CTI by other stratification factors were detected (interaction *p* > 0.05). In sensitivity analyses excluding early deaths within 2 years, individuals with a history of cancer, and those missing covariate data, the findings remained in agreement with the primary analysis (Tables S10–15). After reclassifying CKM into nonadvanced CKM stages, CKM Stage 3, and CKM Stage 4 (Table S16), the overall direction of the associations remained generally consistent with the primary analysis although some stage‐specific heterogeneity was observed. Similarly, in an additional sensitivity analysis using the 5‐level CKM classification (Stages 0–4), higher CTI remained associated with more advanced CKM stages across sequentially adjusted ordinal logistic regression models, and the overall pattern of results was generally consistent with the primary analysis (Table S17). In the parsimonious adjustment analysis excluding cardiometabolic, renal, and medication‐related variables, the associations between CTI and mortality remained consistent with the primary analysis (Table S18). In the overall population, each SD increase in CTI was associated with higher risks of all‐cause mortality (HR = 1.34, 95% CI: 1.25–1.44) and cardiovascular mortality (HR = 1.48, 95% CI: 1.31–1.69), with similar patterns in nonadvanced and advanced CKM stages.

## 4. Discussion

Baseline analyses indicated that higher CTI concentrations were accompanied by a greater prevalence of advanced CKM stages. During 152 median follow‐up months, higher CTI concentrations were significantly associated with higher risks of all‐cause and cardiovascular mortality, with homogeneous associations across various CKM stage groups. Moreover, analyses stratified by subgroups indicated a stronger association between CTI and mortality among participants younger than 60 years. Among individuals who drank alcohol, elevated CTI was associated with higher all‐cause mortality, while no similar association emerged for cardiovascular mortality. Taken together, these findings suggest that CTI is aligned with CKM stage severity and associated with subsequent mortality risk. Moreover, the strength of this association may vary across subgroups (e.g., age and alcohol consumption), highlighting potential heterogeneity in risk.

The distribution of CKM stages in this study also warrants consideration. More than half of the participants were classified as CKM Stage 2, whereas a smaller proportion was classified as Stage 3. This pattern may partly reflect the broad operational definition of Stage 2, which includes diabetes, hypertension, hypertriglyceridemia, MeTS, and moderate‐to‐high‐risk CKD. In contrast, Stage 3 was defined as subclinical CVD and was operationalized mainly using a predicted 10‐year CVD risk ≥ 20% based on the PREVENT equations or very‐high‐risk CKD. Because the PREVENT equations estimate future 10‐year CVD event risk rather than directly detect existing subclinical cardiovascular abnormalities, participants with unmeasured subclinical disease but a predicted risk below this threshold may have remained classified as Stage 2. In addition, the lack of imaging, biomarker, or other diagnostic data in NHANES may have further limited the identification of subclinical CVD. Therefore, the relatively low prevalence of Stage 3 may partly reflect the operational definition and available data rather than the true absence of subclinical cardiovascular disease.

Importantly, the relationship between CTI and CKM stage severity should be interpreted in the context of potential conceptual and methodological overlap. CTI incorporates FBG and TG, whereas CKM staging includes glycemic and lipid‐related metabolic abnormalities, including diabetes, hypertriglyceridemia, MeTS, and obesity‐related metabolic dysfunction. Therefore, this finding should not be interpreted as evidence that CTI is completely independent of the CKM staging framework. Rather, higher CTI may reflect an integrated inflammatory–metabolic burden that is closely aligned with CKM severity. Notably, CRP, the inflammatory component of CTI, is not incorporated into any CKM staging criteria, suggesting that CTI captures an inflammatory dimension that is at least partially complementary to, rather than entirely redundant with, the metabolic components used in CKM classification.

Prior research in general populations has shown that higher baseline TyG index correlates with greater mortality risk [31]. Similar associations were also observed among individuals diagnosed with diabetes, CVD, CKD, and MeTS, each of which is closely associated with CKM syndrome [32–34]. Additionally, systemic inflammation is recognized as another important contributor to mortality risk. Among inflammatory biomarkers, CRP has attracted the greatest attention owing to its application in CVD screening and risk reclassification. Analysis of UK Biobank data indicated that CRP independently predicted subsequent atherothrombotic events [35]. Additionally, a meta‐analysis demonstrated persistent associations between CRP levels and outcomes such as ischemic stroke, coronary events, and vascular deaths, as well as mortality related to pulmonary disease and cancer [36]. However, reliance on TyG or CRP alone captures only one dimension of underlying pathophysiology. Consequently, an increasing number of studies have employed these markers in combination. In the ROC analyses, CTI generally demonstrated higher marker‐level AUCs than TyG and CRP across outcomes and time points. However, the incremental predictive performance analysis showed that the added value of CTI beyond conventional clinical covariates was modest. Adding CTI to the reference clinical covariate model resulted in only small increases in the C statistic for both all‐cause and cardiovascular mortality although NRI and IDI suggested some improvement in risk reclassification and discrimination. Therefore, CTI should be interpreted as a potential inflammatory–metabolic marker associated with mortality risk rather than as a validated clinical prediction tool. One possible explanation is that CTI may provide a broader perspective on overall health status by integrating information related to both inflammation and IR, thereby capturing multiple dimensions of underlying pathological changes. Feng et al. demonstrated that the concurrent inclusion of CRP and the TyG index in traditional risk models significantly enhanced the ability to stratify CVD risk [37]. Cui et al. found that concurrent exposure to elevated TyG index and CRP was significantly correlated with the incidence of high‐risk CVD events, with mutual mediation observed between the two markers [38]. However, comprehensive evidence assessing CTI as a marker of mortality risk in those diagnosed with CKM syndrome remains scarce. Our analysis demonstrated that higher CTI levels were significantly associated with higher risks of both overall and cardiovascular mortality, showing homogeneous associations across different CKM stages.

Although the exact mechanisms linking CTI to mortality risk remained unclear, they could be elucidated from the following perspectives. First, IR induced increased generation of glycation products and free radicals, impaired nitric oxide release and endothelial function, and led to inflammation, oxidative stress, and atherosclerosis, which could have contributed to the development of CVD [39]. Second, IR impaired renal hemodynamics through mechanisms that activated sympathetic nervous system, reduced natriuretic peptide activity, and increased sodium retention, thus promoting the progression of kidney disease [40]. Lastly, IR disrupted the equilibrium among signaling pathways, thereby resulting in metabolic dysfunction [41]. On the other hand, inflammation exerts effects on multiple systems. First, inflammatory activity triggers cytokine secretion such as TNF‐α, IL‐6, and CRP, which promote atherosclerosis, impair endothelial function, and increase vascular stiffness, ultimately causing hypertension and heart failure [42]. Second, inflammation promoted monocyte and macrophage infiltration and renal fibrosis, thereby causing renal dysfunction [43]. Furthermore, inflammation and IR exhibited mutual synergistic effects, each exacerbating the other [44]. These mechanisms encompassed multiple systems, and their interplay exerted a profound effect on mortality that surpassed the simple sum of their individual contributions [6]. Therefore, CTI may help identify individuals at higher risk among patients with CKM syndrome although further studies are needed to clarify its clinical utility.

Analyses stratified by subgroups indicated a stronger association between CTI and mortality among participants younger than 60 years, a finding that aligns with reports from the Kailuan study [45]. This observation may be attributable to the greater propensity of younger individuals to adopt unhealthy lifestyle habits, such as drinking and smoking. A pooled, population‐based cohort study similarly confirmed this finding, revealing that younger adults, compared with their older counterparts, had higher attributable risks associated with hypertension, diabetes, obesity, and current smoking [46]. Furthermore, our analysis indicated that alcohol consumers with higher CTI exhibited an elevated risk of all‐cause mortality, while this pattern did not extend to cardiovascular mortality. A prospective Russian cohort investigation demonstrated that alcohol intake markedly elevated the risk of death [47]. The lack of this association for cardiovascular mortality likely reflects an insufficient number of cardiovascular‐related deaths. Therefore, among individuals under 60 years of age, more proactive preventive measures targeting unhealthy lifestyle habits are warranted to reduce mortality and improve prognosis. Apart from the age and drinking subgroups, no notable interactions emerged between CTI and mortality across the remaining stratifications (gender, race, smoking consumption, diabetes, and hypertension), which indicates the robustness of our findings.

This study offers several advantages. To begin with, this is a large, nationally representative analysis examining CTI levels across CKM stages and mortality risk across CKM stages. Second, CTI was evaluated both as a continuous variable and as a categorical measure standardized by SD, which facilitated the assessment of its relationship with mortality and enabled the detection of risk gradients across CTI strata. Third, we rigorously controlled for potential confounding factors and performed extensive subgroup as well as sensitivity analyses to reinforce the stability and credibility of our findings. Finally, CTI, as a biomarker derived from routinely available measures, may help identify individuals with CKM syndrome who are at higher risk.

Taken together, our findings suggest that CTI may provide complementary information for mortality risk assessment across CKM stages; however, its incremental predictive utility beyond conventional clinical covariates appears modest and requires further validation. Further longitudinal studies with repeated measurements, as well as interventional studies, are warranted to clarify causality and evaluate the clinical utility of CTI.

Nevertheless, this study had several limitations. First, Stage 3 CKM was operationalized using predicted 10‐year CVD risk based on the PREVENT equations or very‐high‐risk CKD, and NHANES lacked imaging, biomarker, or other diagnostic data for subclinical CVD. Therefore, some participants with unmeasured subclinical cardiovascular abnormalities may have been misclassified into lower CKM stages, and the prevalence of Stage 3 and advanced CKM stages may have been underestimated. Second, CTI levels were captured solely at the study baseline, and the absence of longitudinal data precluded evaluation of changes in CTI over time and their association with mortality. Third, although analyses were adjusted for multiple variables, residual and unmeasured confounding factors may still have been present. Fourth, this study used NHANES database and because study population was drawn from the American, the extrapolation of our findings to other populations was limited, thereby necessitating further validation in diverse race groups. Additionally, incorporation bias related to the overlap between CTI components and CKM staging criteria should be considered. CTI includes FBG and TG, whereas CKM staging includes diabetes, hypertriglyceridemia, MeTS, and obesity‐related metabolic dysfunction. Thus, part of the observed association between CTI and advanced CKM stages may reflect shared metabolic components rather than an entirely independent biological relationship. Although the association was attenuated after adjustment for cardiometabolic, renal, medication‐related, and other clinical covariates, it remained statistically significant. Nevertheless, this finding should be interpreted cautiously, and CTI should be regarded as an integrated inflammatory–metabolic marker aligned with CKM severity rather than as a marker fully independent of the CKM staging framework. In addition, participants excluded because of missing CTI‐related variables were broadly comparable to those included in the analysis across most baseline characteristics although some differences in several clinical measures were observed; therefore, potential selection bias cannot be fully excluded. Moreover, some covariates in the fully adjusted model may have dual roles as confounders and potential intermediates. For example, eGFR and UACR may reflect pre‐existing kidney dysfunction but may also partly lie on the pathway from inflammatory–metabolic burden to mortality. Therefore, the fully adjusted estimates should be interpreted as conservative associations after extensive adjustment and may be attenuated relative to the total association of CTI with mortality. Although appropriate NHANES weighting was applied to account for the complex survey design and nonresponse, residual nonresponse bias cannot be fully ruled out. Lastly, as an observational study, our analysis could not confirm a causal link between CTI and mortality risk across CKM Stages 0–4.

## 5. Conclusion

Higher CTI levels were accompanied by a greater prevalence of advanced CKM stages and were associated with increased risks of all‐cause and cardiovascular mortality across CKM stages. CTI may provide complementary information for mortality‐risk assessment although further validation is needed.

## Author Contributions

Siqi Yi contributed to the conception and design of the study, oversaw data acquisition, conducted statistical analyses, and prepared the initial manuscript draft. Yanchen Zhu, Shiping Wu, and He Zheng assisted with data collection. Qiujin Huang offered administrative, technical, and material assistance. Weida Qiu contributed to the development of the study concept and overall design. Yingqing Feng and Ying Wu secured funding and also provided administrative, technical, and material support.

## Funding

This work was supported by the Noncommunicable Chronic Diseases‐National Science and Technology Major Project of China (Grant #No. 2023ZD0508906 and Grant #No. 2024ZD0526803), the Climbing Plan of Guangdong Provincial People’s Hospital (DFJH2020022), Guangdong Special Funds for Science and Technology Innovation Strategy, China (Stability support for scientific research institutions affiliated to Guangdong Province‐GDCI 2024), and The Key Area R&D Program of Guangdong Province (No. 2019B020227005).

## Disclosure

All authors participated in the revision process and provided approval for the final version of the manuscript.

## Ethics Statement

The NHANES protocol was approved by the Ethics Review Board of the National Center for Health Statistics, and all participants provided written informed consent prior to enrollment. This study was conducted in accordance with the Declaration of Helsinki. Since this study used deidentified data from NHANES, no additional ethics approval is required.

## Consent

Please check the Ethics Statement.

## Conflicts of Interest

The authors declare no conflicts of interest.

## Supporting Information

Additional supporting information can be found online in the Supporting Information section.

## Supporting information


**Supporting Information 1** Table S1 shows the baseline characteristics of the included population and those excluded due to missing CTI components. Table S2 shows the specific definitions of diseases used for the staging of cardiovascular–kidney–metabolic syndrome. Table S3 shows the specific definitions of Stages 0–4 of the cardiovascular–kidney–metabolic syndrome. Table S4 shows the formula for calculating 10‐year cardiovascular risk, which is used in the CKM Stage 3. Table S5 shows the missing covariates and the percentage of missing values. Table S6 shows the normality tests for the continuous variables used in the regression analysis. Table S7 examines the multicollinearity of all variables in the models. Table S8 shows the baseline characteristics of the population stratified by CTI. Table S9 shows the discrimination and reclassification statistics for all‐cause and cardiovascular mortality after adding CTI to the reference clinical covariate model. Tables S10–S15 present sensitivity analyses of the primary outcome. Tables S10‐S11 excludes patients who experienced an event within 2 years of follow‐up. Tables S12‐S13 exclude patients with a history of cancer, and Tables S14‐S15 exclude all patients with any missing values. Table S16 shows the association between CTI and all‐cause and cardiovascular mortality stratified by CKM stages (0–2, 3, and 4). Table S17 shows the association between CTI and cardiovascular‐kidney‐metabolic stages (0–4). Table S18 shows the association between CTI and all‐cause and cardiovascular mortality using a parsimonious adjustment model excluding cardiometabolic, renal, and medication‐related variables. Figure S1 shows the inclusion and exclusion flow chart for the study population. Figure S2 uses the restricted cubic splines model to evaluate the nonlinear relationship between CTI and advanced CKM. Figure S3 shows the Kaplan–Meier curves for all‐cause mortality and cardiovascular mortality in different CTI groups. Figure S4 shows the time‐dependent receiver operating characteristic (ROC) curves for all‐cause mortality and cardiovascular mortality at 60 and 120 months.

**Supporting Information 2** STROBE.

## Data Availability

All the datasets collected and analyzed during this study are available on the NHANES website (https://www.cdc.gov/nchs/nhanes/).

## References

[1] Ndumele C. E. , Rangaswami J. , Chow S. L. et al., Cardiovascular-Kidney-Metabolic Health: A Presidential Advisory from the American Heart Association, Circulation. (2023) 148, no. 20, 1606–1635, 10.1161/cir.0000000000001184. 37807924

[2] Jankowski J. , Floege J. , Fliser D. , Bohm M. , and Marx N. , Cardiovascular Disease in Chronic Kidney Disease: Pathophysiological Insights and Therapeutic Options, Circulation. (2021) 143, no. 11, 1157–1172, 10.1161/circulationaha.120.050686. 33720773 PMC7969169

[3] Wilson P. W. , D′Agostino R. B. , Parise H. , Sullivan L. , and Meigs J. B. , Metabolic Syndrome as a Precursor of Cardiovascular Disease and Type 2 Diabetes Mellitus, Circulation. (2005) 112, no. 20, 3066–3072, 10.1161/circulationaha.105.539528. 16275870

[4] Silveira R. J. L. , Barbalho S. M. , Reverete de Araujo R. , Bechara M. D. , Sloan K. P. , and Sloan L. A. , Metabolic Syndrome and Cardiovascular Diseases: Going Beyond Traditional Risk Factors, Diabetes Metab Res Rev. (2022) 38, no. 3, 10.1002/dmrr.3502. 34614543

[5] Scurt F. G. , Ganz M. J. , Herzog C. , Bose K. , Mertens P. R. , and Chatzikyrkou C. , Association of Metabolic Syndrome and Chronic Kidney Disease, Obesity Reviews. (2024) 25, no. 1, 10.1111/obr.13649. 37783465

[6] Ndumele C. E. , Neeland I. J. , Tuttle K. R. et al., A Synopsis of the Evidence for the Science and Clinical Management of cardiovascular-kidney-metabolic (CKM) Syndrome: a Scientific Statement from the American Heart Association, Circulation. (2023) 148, no. 20, 1636–1664, 10.1161/cir.0000000000001186. 37807920

[7] GbdcoD C. , Global Burden of 288 Causes of Death and Life Expectancy Decomposition in 204 Countries and Territories and 811 Subnational Locations, 1990-2021: A Systematic Analysis for the Global Burden of Disease Study 2021, Lancet. (2024) 403, no. 10440, 2100–2132. 38582094 10.1016/S0140-6736(24)00367-2 PMC11126520

[8] Aggarwal R. , Ostrominski J. W. , and Vaduganathan M. , Prevalence of cardiovascular-kidney-metabolic Syndrome Stages in US Adults, 2011-2020, JAMA. (2024) 331, no. 21, 1858–1860, 10.1001/jama.2024.6892. 38717747 PMC11079779

[9] Ostrominski J. W. , Arnold S. V. , Butler J. et al., Prevalence and Overlap of Cardiac, Renal, and Metabolic Conditions in US Adults, 1999-2020, JAMA Cardiology. (2023) 8, no. 11, 1050–1060, 10.1001/jamacardio.2023.3241. 37755728 PMC10535010

[10] Malik S. , Wong N. D. , Franklin S. S et al., Impact of the Metabolic Syndrome on Mortality from Coronary Heart Disease, Cardiovascular Disease, and all Causes in United States Adults, Circulation. (2004) 110, no. 10, 1245–1250, 10.1161/01.cir.0000140677.20606.0e. 15326067

[11] Lopez-Jaramillo P. , Gomez-Arbelaez D. , Martinez-Bello D et al., Association of the Triglyceride Glucose Index as a Measure of Insulin Resistance with Mortality and Cardiovascular Disease in Populations from Five Continents (PURE Study): A Prospective Cohort Study, The Lancet. Healthy Longevity. (2023) 4, no. 1, e23–e33, 10.1016/s2666-7568(22)00247-1. 36521498

[12] Cui H. , Liu Q. , Wu Y. , and Cao L. , Cumulative triglyceride-glucose Index is a Risk for CVD: A Prospective Cohort Study, Cardiovascular Diabetology. (2022) 21, no. 1, 10.1186/s12933-022-01456-1. PMC8830002 35144621

[13] Park H. M. , Lee H. S. , Lee Y. J. , and Lee J. H. , The triglyceride-glucose Index is a More Powerful Surrogate Marker for Predicting the Prevalence and Incidence of Type 2 Diabetes Mellitus than the Homeostatic Model Assessment of Insulin Resistance, Diabetes Research and Clinical Practice. (2021) 180, 10.1016/j.diabres.2021.109042. 34506839

[14] Kunutsor S. K. , Seidu S. , Kurl S. , and Laukkanen J. A. , Baseline and Usual triglyceride-glucose Index and the Risk of Chronic Kidney Disease: A Prospective Cohort Study, GeroScience. (2024) 46, no. 3, 3035–3046, 10.1007/s11357-023-01044-5. 38180700 PMC11009217

[15] Lin H. Y. , Zhang X. J. , Liu Y. M. , Geng L. Y. , Guan L. Y. , and Li X. H. , Comparison of the Triglyceride Glucose Index and Blood Leukocyte Indices as Predictors of Metabolic Syndrome in Healthy Chinese Population, Scientific Reports. (2021) 11, no. 1, 10.1038/s41598-021-89494-9. PMC8113526 33976344

[16] Gao C. , Gao S. , Zhao R. et al., Association Between Systemic immune-inflammation Index and Cardiovascular-Kidney-Metabolic Syndrome, Scientific Reports. (2024) 14, no. 1, 10.1038/s41598-024-69819-0. PMC11333479 39160192

[17] Pepys M. B. and Hirschfield G. M. , C-reactive Protein: a Critical Update, Journal of Clinical Investigation. (2003) 111, no. 12, 1805–1812. 12813013 10.1172/JCI18921 PMC161431

[18] Ruan G. T. , Xie H. L. , Zhang H. Y. et al., A Novel Inflammation and Insulin Resistance Related Indicator to Predict the Survival of Patients With Cancer, Frontiers in Endocrinology. (2022) 13, 10.3389/fendo.2022.905266. PMC9252441 35795140

[19] Shan Y. , Liu Q. , and Gao T. , Application of the C-Reactive Protein-Triglyceride Glucose Index in Predicting the Risk of New-Onset Diabetes in the General Population Aged 45 Years and Older: A National Prospective Cohort Study, BMC Endocrine Disorders. (2025) 25, no. 1, 10.1186/s12902-025-01947-8. PMC11765927 39865224

[20] Huo G. , Tang Y. , Liu Z. , Cao J. , Yao Z. , and Zhou D. , Association Between C-Reactive Protein-Triglyceride Glucose Index and Stroke Risk in Different Glycemic Status: Insights From the China Health and Retirement Longitudinal Study (CHARLS), Cardiovascular Diabetology. (2025) 24, no. 1, 10.1186/s12933-025-02686-9. PMC11948880 40140859

[21] Xu M. , Zhang L. , Xu D. , Shi W. , and Zhang W. , Usefulness of C-Reactive Protein-Triglyceride Glucose Index in Detecting Prevalent Coronary Heart Disease: Findings from the National Health and Nutrition Examination Survey 1999-2018, Frontiers in Cardiovascular Medicine. (2024) 11, 10.3389/fcvm.2024.1485538. PMC11518723 39473894

[22] Zhao D. F. , Value of C-Reactive Protein-Triglyceride Glucose Index in Predicting Cancer Mortality in the General Population: Results from National Health and Nutrition Examination Survey, Nutrition and Cancer. (2023) 75, no. 10, 1934–1944, 10.1080/01635581.2023.2273577. 37873764

[23] Wang Y. , Yang Y. , Chen J. et al., Transition of BMI Status from Childhood to Adulthood and cardiovascular-kidney-metabolic Syndrome in Midlife: A 36-Year Cohort Study, Diabetes Care. (2025) 48, no. 12, 2045–2053, 10.2337/dca25-0027. 40674006

[24] Ding X. , Tian J. , Chang X. , Liu J. , and Wang G. , Association Between Remnant Cholesterol and the Risk of Cardiovascular-Kidney-Metabolic Syndrome Progression: Insights From the China Health and Retirement Longitudinal Study, European Journal of Preventive Cardiology. (2025) 32, no. 13, 1157–1165, 10.1093/eurjpc/zwaf248. 40272444

[25] Khan S. S. , Matsushita K. , Sang Y. et al., Development and Validation of the American Heart Association′s PREVENT Equations, Circulation. (2024) 149, no. 6, 430–449, 10.1161/circulationaha.123.067626. 37947085 PMC10910659

[26] Madero M. , Levin A. , Ahmed S. B. et al., Evaluation and Management of Chronic Kidney Disease: Synopsis of the Kidney Disease: Improving Global Outcomes 2024 Clinical Practice Guideline, Annals of internal medicine. (2025) 178, no. 5, 705–713, 10.7326/annals-24-01926. 40063957

[27] Inker L. A. , Eneanya N. D. , Coresh J. et al., New Creatinine- and Cystatin C-based Equations to Estimate GFR Without Race, New England Journal of Medicine. (2021) 385, no. 19, 1737–1749, 10.1056/nejmoa2102953. 34554658 PMC8822996

[28] Yim Y. , Lee J. E. , Son Y. et al., Long-Term Trends in the Prevalence of Cardiovascular-Kidney-Metabolic Syndrome in South Korea, 2011-2021: A Representative Longitudinal Serial Study, Lancet Regional Health. Western Pacific. (2025) 55, 10.1016/j.lanwpc.2025.101474. PMC11795540 39911647

[29] Zhang P. , Mo D. , Zeng W. , and Dai H. , Association Between Triglyceride-Glucose Related Indices and all-Cause and Cardiovascular Mortality Among the Population With Cardiovascular-Kidney-Metabolic Syndrome Stage 0-3: A Cohort Study, Cardiovascular Diabetology. (2025) 24, no. 1, 10.1186/s12933-025-02642-7. PMC11871745 40022225

[30] Zhu R. , Wang R. , He J et al., Prevalence of Cardiovascular-Kidney-Metabolic Syndrome Stages by Social Determinants of Health, JAMA Network Open. (2024) 7, no. 11, 10.1001/jamanetworkopen.2024.45309. PMC11574692 39556396

[31] Chen J. , Wu K. , Lin Y. , Huang M. , and Xie S. , Association of Triglyceride Glucose Index With All-Cause and Cardiovascular Mortality in the General Population, Cardiovascular Diabetology. (2023) 22, no. 1, 10.1186/s12933-023-02054-5. PMC10666367 37993902

[32] Wei X. , Min Y. , Song G. , Ye X. , and Liu L. , Association Between Triglyceride-Glucose Related Indices With the all-Cause and Cause-specific Mortality Among the Population with Metabolic Syndrome, Cardiovascular Diabetology. (2024) 23, no. 1, 10.1186/s12933-024-02215-0. PMC11044377 38658993

[33] Ye Z. , An S. , Gao Y. et al., Association Between the Triglyceride Glucose Index and in-hospital and 1-Year Mortality in Patients With Chronic Kidney Disease and Coronary Artery Disease in the Intensive Care Unit, Cardiovascular Diabetology. (2023) 22, no. 1, 10.1186/s12933-023-01843-2. PMC10183125 37179310

[34] Zhang Q. , Xiao S. , Jiao X. , and Shen Y. , The Triglyceride-Glucose Index is a Predictor for Cardiovascular and All-Cause Mortality in CVD Patients With Diabetes or Pre-Diabetes: Evidence From NHANES 2001–2018, Cardiovascular Diabetology. (2023) 22, no. 1, 10.1186/s12933-023-02030-z. PMC10583314 37848879

[35] Markus M. R. P. , Ittermann T. , Marino Coronado J. et al., LDL-Cholesterol, Lipoprotein(A) and High-Sensitivity Low-Density Lipoprotein Cholesterol, Lipoprotein(A) and High-Sensitivity C-Reactive Protein are Independent Predictors of Cardiovascular Events, European Heart Journal. (2025) . 10.1093/eurheartj/ehaf281 PMC12517743 40320753

[36] Emerging Risk Factors C. , Kaptoge S. , Di Angelantonio E. et al., C-Reactive Protein Concentration and Risk of Coronary Heart Disease, Stroke, and Mortality: An Individual Participant Meta-Analysis, Lancet. (2010) 375, no. 9709, 132–140. 20031199 10.1016/S0140-6736(09)61717-7 PMC3162187

[37] Feng G. , Yang M. , Xu L et al., Combined Effects of High Sensitivity C-Reactive Protein and Triglyceride-Glucose Index on Risk of Cardiovascular Disease Among middle-Aged and Older Chinese: Evidence From the China Health and Retirement Longitudinal Study, Nutrition, Metabolism, and Cardiovascular Diseases. (2023) 33, no. 6, 1245–1253, 10.1016/j.numecd.2023.04.001. 37095018

[38] Cui C. , Liu L. , Qi Y. et al., Joint Association of Tyg Index and High Sensitivity C-Reactive Protein With Cardiovascular Disease: A National Cohort Study, Cardiovascular Diabetology. (2024) 23, no. 1, 10.1186/s12933-024-02244-9. PMC11077847 38715129

[39] Molina M. N. , Ferder L. , and Manucha W. , Emerging Role of Nitric Oxide and Heat Shock Proteins in Insulin Resistance, Current Hypertension Reports. (2016) 18, no. 1, 10.1007/s11906-015-0615-4. 26694820

[40] Spoto B. , Pisano A. , and Zoccali C. , Insulin Resistance in Chronic Kidney Disease: A Systematic Review, American Journal of Physiology- enal Physiology. (2016) 311, no. 6, F1087–F1108, 10.1152/ajprenal.00340.2016. 27707707

[41] Pei J. , Wang B. , and Wang D. , Current Studies on Molecular Mechanisms of Insulin Resistance, Journal of Diabetes Research. (2022) 2022, 1863429–11, 10.1155/2022/1863429. 36589630 PMC9803571

[42] Libby P. , Ridker P. M. , and Hansson G. K. , Leducq Transatlantic Network on A: IBflammation in Atherosclerosis: from Pathophysiology to practice, Journal of the American College of Cardiology. (2009) 54, no. 23, 2129–2138. 19942084 10.1016/j.jacc.2009.09.009 PMC2834169

[43] Tonelli M. , Sacks F. , Pfeffer M. , Jhangri G. S. , and Curhan G. , Cholesterol, Recurrent Events Trial I: BBomarkers of Inflammation and Progression of Chronic Kidney disease, Kidney International. (2005) 68, no. 1, 237–245. 15954913 10.1111/j.1523-1755.2005.00398.x

[44] Shoelson S. E. , Lee J. , and Goldfine A. B. , Inflammation and Insulin Resistance, Journal of Clinical Investigation. (2006) 116, no. 7, 1793–1801. 16823477 10.1172/JCI29069 PMC1483173

[45] Li N. , Li Y. , Cui L. et al., Association Between Different Stages of Cardiovascular-Kidney-Metabolic Syndrome and the Risk of all-Cause Mortality, Atherosclerosis. (2024) 397, 10.1016/j.atherosclerosis.2024.118585. 39255681

[46] Tromp J. , Paniagua S. M. A. , Lau E. S. et al., Age Dependent Associations of Risk Factors With Heart Failure: Pooled Population Based Cohort Study, BMJ. (2021) 372, 10.1136/bmj.n461. PMC7986583 33758001

[47] Leon D. A. , Saburova L. , Tomkins S. et al., Hazardous Alcohol Drinking and Premature Mortality in Russia: A Population Based Case-Control Study, Lancet. (2007) 369, no. 9578, 2001–2009, 10.1016/s0140-6736(07)60941-6. 17574092

