# Supplementary material for: Association Between C‐Reactive Protein–Triglyceride Glucose Index and All‐Cause and Cardiovascular Mortality Across Cardiovascular–Kidney–Metabolic Syndrome Stages 0–4
Source: Int J Endocrinol. 2026 Jul 30;2026:6690501. doi: 10.1155/ije/6690501 (PMC13422643; doi:10.1155/ije/6690501)
Supplement: Supplementary file 1 — Supporting Information 1 Table S1 shows the baseline characteristics of the included population and those excluded due to missing CTI components. Table S2 shows the specific definitions of diseases used for the staging of cardiovascular–kidney–metabolic syndrome. Table S3 shows the specific definitions of Stages 0–4 of the cardiovascular–kidney–metabolic syndrome. Table S4 shows the formula for calculating 10‐year cardiovascular risk, which is used in the CKM Stage 3. Table S5 shows the missing covariates and the percentage of missing values. Table S6 shows the normality tests for the continuous variables used in the regression analysis. Table S7 examines the multicollinearity of all variables in the models. Table S8 shows the baseline characteristics of the population stratified by CTI. Table S9 shows the discrimination and reclassification statistics for all‐cause and cardiovascular mortality after adding CTI to the reference clinical covariate model. Tables S10–S15 present sensitivity analyses of the primary outcome. Tables S10‐S11 excludes patients who experienced an event within 2 years of follow‐up. Tables S12‐S13 exclude patients with a history of cancer, and Tables S14‐S15 exclude all patients with any missing values. Table S16 shows the association between CTI and all‐cause and cardiovascular mortality stratified by CKM stages (0–2, 3, and 4). Table S17 shows the association between CTI and cardiovascular‐kidney‐metabolic stages (0–4). Table S18 shows the association between CTI and all‐cause and cardiovascular mortality using a parsimonious adjustment model excluding cardiometabolic, renal, and medication‐related variables. Figure S1 shows the inclusion and exclusion flow chart for the study population. Figure S2 uses the restricted cubic splines model to evaluate the nonlinear relationship between CTI and advanced CKM. Figure S3 shows the Kaplan–Meier curves for all‐cause mortality and cardiovascular mortality in different CTI groups. Figure S4 shows the [file IJE-2026-6690501-s002.docx]

**Table S1.** Baseline characteristics of Included, and CTI Missing Excluded Population

**Table S2.** Specific Definitions of Various Diseases

**Table S3.** Definition of CKM Syndrome

**Table S4.** The Basic PREVENT 10-Year Risk Estimation Model Equations

**Table S5.** Proportion of Variables with Missing Data

**Table S6.** Normality Test for Continuous Variables

**Table S7.** Collinearity Statistics

**Table S8.** Baseline characteristics of participants classified by CTI quartiles

**Table S9.** Discrimination and reclassification statistics (95% CI) for all-cause and cardiovascular mortality after addition of CTI to the reference clinical covariate model

**Table S10.** Association between CTI and advanced cardiovascular-kidney-metabolic stages excluding events occurred in the first two year of follow up

**Table S11.** Association between CTI and All-Cause and cardiovascular Mortality stratified by Advanced CKM stages excluding events occurred in the first two year of follow up

**Table S12.** Association between CTI and advanced cardiovascular-kidney-metabolic stages excluding individuals with a history of cancer

**Table S13.** Association between CTI and All-Cause and cardiovascular Mortality stratified by Advanced CKM stages excluding individuals with a history of cancer

**Table S14.** Association between CTI and advanced cardiovascular-kidney-metabolic stages excluding those with any missing values

**Table S15.** Association between CTI and All-Cause and cardiovascular Mortality stratified by Advanced CKM stages excluding those with any missing values

**Table S16.** Association between CTI and All-Cause and cardiovascular Mortality stratified by CKM stages (0-2, 3, 4)

**Table S17.** Association between CTI and cardiovascular-kidney-metabolic stages (0-4)

**Table S18.** Association between CTI and All-Cause and cardiovascular Mortality using a parsimonious adjustment model

**Figure S1**. Participant Selection Process Flowchart

**Figure S2.** Restricted cubic splines illustrate the relationship between CTI and advanced CKM stages.

**Figure S3.**Kaplan–Meier analyses for all-cause (A) and cardiovascular mortality (B) across different C-reactive protein-triglyceride glucose index groups

**Figure S4.** Time-dependent receiver operating characteristic (ROC) curves for all-cause mortality and cardiovascular mortality at 60 and 120 months

**Table S1. Baseline characteristics of Included, and CTI Missing Excluded Population**

| **Variables** | **Included population** | **CTI Missing Excluded Population** | ***P*-value** |
| --- | --- | --- | --- |
| Total | 8314 | 9341 |  |
| Age, years | 49.00 (35.00, 64.00) | 49.00 (35.00, 64.00) | 0.301 |
| Gender, Female, n (%) | 3985 (47.9) | 4505 (48.2) | 0.705 |
| Race, n (%) |  |  | 0.211 |
| Mexican American | 1750 (21.0) | 1942 (20.8) |  |
| Other Hispanic | 649 (7.8) | 649 (6.9) |  |
| Non-Hispanic White | 4249 (51.1) | 4830 (51.7) |  |
| Non-Hispanic Black | 1359 (16.3) | 1550 (16.6) |  |
| Other Race | 307 (3.7) | 370 (4.0) |  |
| PIR | 2.34 (1.24, 4.32) | 2.34 (1.20, 4.29) | 0.078 |
| Marital, n (%) | 5345 (64.3) | 5829 (63.3) | 0.256 |
| Education, n (%) |  |  | 0.506 |
| Less than high school | 2419 (29.1) | 2783 (29.8) |  |
| High school or equivalent | 1969 (23.7) | 2204 (23.6) |  |
| College or above | 3926 (47.2) | 4343 (46.5) |  |
| Smoking, n (%) |  |  | 0.122 |
| Never | 4324 (52.0) | 4800 (51.4) |  |
| Former | 2218 (26.7) | 2428 (26.0) |  |
| Current | 1772 (21.3) | 2107 (22.6) |  |
| Drinking, n (%) | 6082 (73.2) | 6397 (72.6) | 0.368 |
| BMI, kg/m^2^ | 27.45 (24.33, 31.12) | 27.60 (24.36, 31.25) | 0.190 |
| Waist circumference, cm | 96.50 (87.60, 105.90) | 96.60 (87.30, 106.00) | 0.673 |
| SBP, mmHg | 120.67 (110.67, 133.33) | 121.33 (112.00, 134.00) | <0.001 |
| DBP, mmHg | 70.00 (63.33, 77.33) | 71.33 (64.00, 78.67) | <0.001 |
| HbA1c, % | 5.40 (5.20, 5.70) | 5.40 (5.20, 5.70) | 0.795 |
| TC, mg/dL | 196.00 (172.00, 224.00) | 198.00 (173.00, 225.00) | 0.095 |
| HDL, mg/dL | 51.00 (42.00, 62.00) | 49.00 (41.00, 60.00) | <0.001 |
| LDL, mg/dL | 118.00 (96.00, 142.00) | 107.00 (95.00, 119.00) | 0.185 |
| UA, mg/dL | 5.40 (4.50, 6.40) | 5.30 (4.40, 6.30) | <0.001 |
| UACR, mg/g | 6.40 (4.10, 12.10) | 6.60 (4.20, 12.30) | 0.148 |
| eGFR, mL/min/1.73 m² | 98.10 (82.60, 112.00) | 96.40 (80.40, 111.10) | <0.001 |
| Antihypertensive drugs, n (%) | 2206 (26.5) | 2437 (26.2) | 0.500 |
| Antihyperglycemic drugs, n (%) | 674 (8.1) | 771 (8.3) | 0.723 |
| Statin use, n (%) | 1537 (18.5) | 1737 (18.6) | 0.868 |
| Liver disease, n (%) | 288 (3.5) | 303 (3.2) | 0.460 |
| Cancer, n (%) | 744 (8.9) | 805 (8.6) | 0.458 |

Abbreviations: CTI, C-reactive protein-triglyceride glucose index; PIR, ratio of family income to poverty; BMI, body mass index; SBP, systolic blood pressure; DBP, diastolic blood pressure; HbA1c, hemoglobin A1c; TC, total cholesterol; HDL, high density lipoprotein cholesterol; LDL, low density lipoprotein cholesterol; UA, uric acid; UACR, urine albumin-to-creatinine ratio; eGFR, estimated glomerular filtration rate;

Normality was assessed using the Kolmogorov-Smirnov test, and all continuous variables were non-normally distributed, thus described as median (interquartile range); categorical variables were expressed as frequency (percentage)

**Table S2. Specific Definitions of Various Diseases**

| Overweight/obesity | BMI ≥ 25 kg/m^2^ (or ≥ 23 kg/m^2^ if Asian ancestry) |
| --- | --- |
| Abdominal obesity | Waist circumference ≥ 88/102 cm in female/male (or if Asian ancestry ≥80/90 cm in female/male) |
| Prediabetes | Fasting blood glucose ≥ 100-125 mg/dL or HbA1c ≥ 5.7%-6.4% and  without self-reported diagnosis of diabetes, use of antihyperglycemic drugs |
| Diabetes | Fasting blood glucose ≥ 126 mg/dL or HbA1c ≥ 6.5% or self-reported diagnosis of diabetes or use of antihyperglycemic drugs |
| Hypertension | SBP ≥130 mm Hg or DBP ≥80 mm Hg or self-reported diagnosis of hypertension or use of antihypertensive drugs |
| chronic kidney disease | Moderate-to-high-risk CKD in KDIGO classification: UACR ≥ 30 mg/g  and eGFR ≥ 60 ml/min/1.73m^2^, UACR < 300 mg/g and eGFR ≤ 45-59  ml/min/1.73m^2^, or UACR < 30 mg/g and eGFR ≤ 30-44 ml/min/1.73m^2^. |
| Hypertriglyceridemia | Triglycerides ≥ 135 mg/dL |
| Metabolic syndrome | Meet any three or more of the five   1. Waist circumference ≥ 88/102cm in female/male. 2. HDL ≤ 50/40 mg/dL in female/male. 3. Triglycerides ≥ 150 mg/dL. 4. Elevated blood pressure (SBP ≥ 130 mm Hg or DBP ≥ 80 mm Hg and/or use of antihypertensive drugs) 5. Prediabetes |
| Clinical CVD | Self-reported established cardiovascular disease (coronary heart disease, angina, heart attack, heart failure, and stroke) |
| Subclinical CVD | Any of the two criteria is met：   1. Very high-risk CKD in KDIGO classification: UACR ≥ 300 mg/g and   eGFR ≤ 45-59 ml/min/1.73m^2^, UACR ≥ 30 mg/g and eGFR ≤ 30-44  ml/min/1.73m^2^, or eGFR ≤ 29 ml/min/1.73m^2^   1. Predicted 10-year CVD risk ≥ 20% |

Abbreviations: BMI, body mass index; HbA1c, hemoglobin A1c; SBP, systolic blood pressure; DBP, diastolic blood pressure; CKD, chronic kidney disease; KDIGO, kidney disease improving global outcomes; UACR, urine albumin-to-creatinine ratio; eGFR, estimated glomerular filtration rate; HDL, high density lipoprotein cholesterol; CVD, cardiovascular diseases

*Asian was not listed as a separate race/ethnicity until NHANES 2011-2012, therefore the uniform threshold for BMI and waist circumference was used in all participants in NHANES 1999-2010

**Table S3. Definition of CKM Syndrome**

| **CKM stages** | **Definition** |
| --- | --- |
| CKM stage 0 | Participants with a normal Body Mass Index (BMI) (<25 kg/m^2^ or <23 kg/m^2^ if Asian ancestry), and a normal waist circumference (<88/102 cm in female/male or if Asian ancestry <80/90 cm in female/male) who did not fulfill the criteria for the other stages. |
| CKM stage 1 | Elevated BMI (≥25 kg/m^2^ or ≥23 kg/m^2^ if Asian ancestry), increased waist circumference (≥88/102 cm in female/male or if Asian ancestry≥80/90 cm in female/male), or prediabetes. Prediabetes is defined as a fasting blood glucose≥100-125 mg/dL or a glycated hemoglobin (HbA1c)≥5.7%-6.4% and without self-reported diagnosis of diabetes, use of antihyperglycemic drugs |
| CKM stage 2 | Metabolic risk factors or moderate-to-high-risk chronic kidney disease (CKD) as defined by the Kidney Disease Improving Global Outcomes (KDIGO) criteria, in accordance with AHA recommendations. The qualifying metabolic risk factors encompassed: elevated fasting serum triglycerides (≥135 mg/dL); hypertension; diabetes; metabolic syndrome, characterized by the presence of at least three of the following:  increased waist circumference; reduced high-density lipoprotein (HDL) cholesterol levels (<40 mg/dL for men, <50 mg/dL for women); fasting serum triglycerides ≥150 mg/dL; elevated blood pressure (systolic ≥130 mmHg, diastolic ≥80 mmHg, and/or use of antihypertensive drugs); prediabetes. |
| CKM stage 3 | Presence of very-high-risk KDIGO CKD stages or a high-estimated 10-year cardiovascular disease (CVD) risk. The 10-year CVD risk was assessed using the AHA PREVENT equations for predicting cardiovascular events. High risk was defined as ≥20% 10-year CVD risk. |
| CKM stage 4 | Self-reported established cardiovascular disease (coronary heart disease, angina, heart attack, heart failure, and stroke). |

Abbreviations: CKM Syndrome, Cardiovascular-Kidney-Metabolic syndrome

**Table S4. The Basic PREVENT 10-Year Risk Estimation Model Equations**

| **10-year CVD risk assessment equation** | |
| --- | --- |
| **Men** | **log-Odds** = -3.031168 + 0.7688528 × (age – 55) /10 + 0.0736174 × ((TC – HDL) × 0.02586 – 3.5) – 0.0954431 × (HDL × 0.02586 – 1.3) /0.3 – 0.4347345 × (min(SBP, 110) – 110) /20 + 0.3362658 × (max(SBP, 110) – 130) /20 + 0.7692857 × (if diabetes) + 0.4386871 × (if current smoker) + 0.5378979 × (min(eGFR, 60) – 60) / -15 + 0.0164827 × (max(eGFR, 60) – 90) / -15 + 0.288879 × (if using anti-hypertensive medication) – 0.1337349 × (if using statin) – 0.0475924 × (if using anti-hypertensive medication) × (max(SBP, 110) – 130) /20 + 0.150273 × (if using statin) × ((TC – HDL) × 0.02586 – 3.5) – 0.0517874 × (age – 55) /10 × ((TC – HDL) × 0.02586 – 3.5) + 0.0191169 × (age – 55) /10 × (HDL × 0.02586 – 1.3) /0.3 – 0.1049477 × (age – 55) /10 × (max(SBP, 110) – 130) /20 – 0.2251948 × (age – 55) /10 × (if diabetes) – 0.0895067 × (age – 55) /10 × (if current smoker) – 0.1543702 × (age – 55) /10 × (min(eGFR, 60) – 60) / -15 |
|  | **Risk** = exp(log-Odds) / (1 + exp(log-Odds)) |
| **Women** | log-Odds = -3.307728 + 0.7939329 × (age – 55) /10 + 0.0305239 × ((TC – HDL) × 0.02586 – 3.5) – 0.1606857 × (HDL × 0.02586 – 1.3) /0.3 – 0.2394003 × (min(SBP, 110) – 110) /20 + 0.360078 × (max(SBP, 110) – 130) /20 + 0.8667604 × (if diabetes) + 0.5360739 × (if current smoker) + 0.6045917 × (min(eGFR, 60) – 60) / -15 + 0.0433769 × (max(eGFR, 60) – 90) / -15 + 0.3151672 × (if using anti-hypertensive medication) – 0.1477655 × (if using statin) – 0.0663612 × (if using anti-hypertensive medication) × (max(SBP, 110) – 130) /20 + 0.1197879 × (if using statin) × ((TC – HDL) × 0.02586 – 3.5) – 0.0819715 × (age – 55) /10 × ((TC – HDL) × 0.02586 – 3.5) + 0.0306769 × (age – 55) /10 × (HDL × 0.02586 × 0.02586 – 1.3) /0.3 – 0.0946348 × (age – 55) /10 × (max(SBP, 110) – 130) /20 – 0.27057 × (age – 55) /10 × (if diabetes) – 0.078715 × (age – 55) /10 × (if current smoker) – 0.1637806 × (age – 55) /10 × (min(eGFR, 60) – 60) / -15 |
|  | **Risk** = exp(log-Odds) / (1 + exp(log-Odds)) |

Abbreviations: TC, total cholesterol; HDL, high-density lipoprotein cholesterol; SBP, systolic blood pressure; eGFR, estimated glomerular filtration rate.

**Table S5. Proportion of Variables with Missing Data**

| **Variables** | **Number of Missing** | **Missing proportion** |
| --- | --- | --- |
| PIR | 660 | 7.9% |
| Drinking | 356 | 4.2% |
| LDL | 201 | 2.4% |
| Marital | 136 | 1.6% |
| Waist circumference | 90 | 1.0% |
| UACR | 49 | 0.5% |
| Antihypertensive drugs | 42 | 0.5% |
| Liver disease | 23 | 0.2% |
| HbA1c | 20 | 0.2% |
| Education | 11 | 0.1% |
| Smoking | 10 | 0.1% |
| Cancer | 9 | 0.1% |
| Antihyperglycemic drugs | 4 | 0.1% |

Abbreviations: PIR, ratio of family income to poverty; LDL, low density lipoprotein cholesterol; UACR, urine albumin-to-creatinine ratio; HbA1c, hemoglobin A1c

**Table S6. Normality Test for Continuous Variables**

| **Kolmogorov-Smirnov** | **Statistic** | ***P*-value** |
| --- | --- | --- |
| Age | 0.058 | <0.001 |
| PIR | 0.120 | <0.001 |
| BMI | 0.041 | <0.001 |
| Waist circumference | 0.016 | <0.001 |
| SBP | 0.088 | <0.001 |
| DBP | 0.043 | <0.001 |
| FBG | 0.237 | <0.001 |
| HbA1c | 0.230 | <0.001 |
| TG | 0.158 | <0.001 |
| TC | 0.045 | <0.001 |
| HDL | 0.073 | <0.001 |
| LDL | 0.040 | <0.001 |
| CRP | 0.311 | <0.001 |
| UA | 0.040 | <0.001 |
| UACR | 0.453 | <0.001 |
| eGFR | 0.047 | <0.001 |

Abbreviations: PIR, ratio of family income to poverty; BMI, body mass index; SBP, systolic blood pressure; HbA1c, hemoglobin A1c; HDL, high density lipoprotein cholesterol; LDL, low density lipoprotein cholesterol; UA, uric acid; UACR, urine albumin-to-creatinine ratio; eGFR, estimated glomerular filtration rate;

**Table S7. Collinearity Statistics**

|  | **GVIF** | **Df** | **GVIF1/2Df** |
| --- | --- | --- | --- |
| CTI | 1.7 | 1 | 1.3 |
| Age | 3.1 | 1 | 1.8 |
| Gender | 1.6 | 1 | 1.3 |
| Race | 1.6 | 4 | 1.1 |
| Marital | 1.1 | 1 | 1.1 |
| Education | 1.5 | 2 | 1.1 |
| Smoking | 1.3 | 2 | 1.1 |
| Drinking | 1.2 | 1 | 1.1 |
| PIR | 1.4 | 1 | 1.2 |
| BMI | 1.4 | 1 | 1.2 |
| SBP | 1.5 | 1 | 1.2 |
| HDL | 1.5 | 1 | 1.2 |
| LDL | 1.1 | 1 | 1.0 |
| HbA1c | 1.8 | 1 | 1.3 |
| UA | 1.7 | 1 | 1.3 |
| eGFR | 2.5 | 1 | 1.6 |
| UACR | 1.1 | 1 | 1.0 |
| Statin use | 1.3 | 1 | 1.1 |
| Antihypertensive drugs | 1.5 | 1 | 1.2 |
| Antihyperglycemic drugs | 1.6 | 1 | 1.3 |
| Liver disease | 1.0 | 1 | 1.0 |
| Cancer | 1.1 | 1 | 1.1 |

Abbreviations: CTI, C-reactive protein-triglyceride glucose index; PIR, ratio of family income to poverty; BMI, body mass index; SBP, systolic blood pressure; HDL, high density lipoprotein cholesterol; LDL, low density lipoprotein cholesterol; HbA1c, hemoglobin A1c; UA, uric acid; eGFR, estimated glomerular filtration rate; UACR, urine albumin-to-creatinine ratio

**Table S8. Baseline characteristics of participants classified by CTI quartiles**

| **Variables** | **Overall** | **Q1 (2.76, 4.49)** | **Q2 (4.49, 4.94)** | **Q3 (4.94, 5.38)** | **Q4 (5.38, 7.39)** | ***P*-value** |
| --- | --- | --- | --- | --- | --- | --- |
| Total | 8314 | 2079 | 2078 | 2078 | 2079 |  |
| Age, years | 49.00 (35.00, 64.00) | 40.00 (29.00, 54.00) | 49.00 (35.25, 64.00) | 53.00 (39.00, 67.00) | 56.00 (42.00, 68.00) | <0.001 |
| Gender, Female, n (%) | 3985 (47.9) | 978 (47.0) | 911 (43.8) | 979 (47.1) | 1117 (53.7) | <0.001 |
| Race, n (%) |  |  |  |  |  | <0.001 |
| Mexican American | 1750 (21.0) | 335 (16.1) | 410 (19.7) | 463 (22.3) | 542 (26.1) |  |
| Other Hispanic | 649 (7.8) | 153 (7.4) | 139 (6.7) | 187 (9.0) | 170 (8.2) |  |
| Non-Hispanic White | 4249 (51.1) | 1063 (51.1) | 1103 (53.1) | 1043 (50.2) | 1040 (50.0) |  |
| Non-Hispanic Black | 1359 (16.3) | 418 (20.1) | 353 (17.0) | 310 (14.9) | 278 (13.4) |  |
| Other Race | 307 (3.7) | 110 (5.3) | 73 (3.5) | 75 (3.6) | 49 (2.4) |  |
| PIR | 2.34 (1.24, 4.32) | 2.75 (1.37, 4.80) | 2.55 (1.32, 4.54) | 2.26 (1.24, 4.13) | 1.97 (1.09, 3.77) | <0.001 |
| Marital, n (%) | 5345 (64.3) | 1284 (61.8) | 1359 (65.4) | 1362 (65.5) | 1340 (64.5) | 0.039 |
| Education, n (%) |  |  |  |  |  | <0.001 |
| Less than high school | 2419 (29.1) | 429 (20.6) | 568 (27.3) | 668 (32.1) | 754 (36.3) |  |
| High school or equivalent | 1969 (23.7) | 426 (20.5) | 513 (24.7) | 518 (24.9) | 512 (24.6) |  |
| College or above | 3926 (47.2) | 1224 (58.9) | 997 (48.0) | 892 (42.9) | 813 (39.1) |  |
| Smoking, n (%) |  |  |  |  |  | <0.001 |
| Never | 4324 (52.0) | 1237 (59.5) | 1101 (53.0) | 1045 (50.3) | 941 (45.3) |  |
| Former | 2218 (26.7) | 458 (22.0) | 551 (26.5) | 591 (28.4) | 618 (29.7) |  |
| Current | 1772 (21.3) | 384 (18.5) | 426 (20.5) | 442 (21.3) | 520 (25.0) |  |
| Drinking, n (%) | 6082 (73.2) | 1601 (77.0) | 1556 (74.9) | 1491 (71.8) | 1434 (69.0) | <0.001 |
| BMI, kg/m^2^ | 27.45 (24.33, 31.12) | 24.48 (22.22, 27.14) | 27.31 (24.46, 30.27) | 28.44 (25.46, 31.83) | 30.08 (26.87, 33.75) | <0.001 |
| Waist circumference, cm | 96.50 (87.60, 105.90) | 87.30 (79.60, 95.00) | 95.80 (88.10, 104.30) | 99.10 (91.50, 107.00) | 103.40 (95.60, 111.80) | <0.001 |
| SBP, mmHg | 120.67 (110.67, 133.33) | 114.67 (106.67, 124.67) | 120.67 (110.67, 132.00) | 122.67 (112.67, 136.00) | 125.33 (114.67, 138.00) | <0.001 |
| DBP, mmHg | 70.00 (63.33, 77.33) | 68.67 (62.00, 75.33) | 70.00 (63.33, 77.33) | 71.33 (64.00, 78.00) | 71.33 (63.33, 78.67) | <0.001 |
| FBG, mg/dL | 98.80 (92.00, 107.60) | 94.00 (88.80, 100.35) | 97.50 (91.10, 105.00) | 99.60 (93.00, 108.35) | 105.00 (96.00, 121.25) | <0.001 |
| HbA1c, % | 5.40 (5.20, 5.70) | 5.30 (5.00, 5.50) | 5.40 (5.20, 5.70) | 5.50 (5.20, 5.80) | 5.60 (5.30, 6.10) | <0.001 |
| TG, mg/dL | 107.00 (74.00, 158.00) | 69.00 (53.00, 90.00) | 97.00 (73.00, 131.00) | 124.00 (92.00, 166.00) | 169.00 (123.00, 238.50) | <0.001 |
| TC, mg/dL | 196.00 (172.00, 224.00) | 184.00 (163.00, 209.00) | 194.00 (171.00, 221.00) | 200.00 (176.00, 228.00) | 208.00 (183.00, 237.00) | <0.001 |
| HDL, mg/dL | 51.00 (42.00, 62.00) | 59.00 (49.00, 69.00) | 52.00 (44.00, 62.00) | 49.00 (41.00, 59.00) | 44.00 (37.00, 53.00) | <0.001 |
| LDL, mg/dL | 118.00 (96.00, 142.00) | 109.00 (90.00, 130.00) | 118.00 (96.00, 141.00) | 122.00 (100.00, 146.00) | 124.00 (100.00, 149.00) | <0.001 |
| CRP, mg/dL | 0.19 (0.08, 0.42) | 0.05 (0.03, 0.08) | 0.14 (0.09, 0.21) | 0.28 (0.19, 0.42) | 0.69 (0.41, 1.15) | <0.001 |
| UA, mg/dL | 5.40 (4.50, 6.40) | 4.90 (4.10, 5.80) | 5.40 (4.50, 6.30) | 5.60 (4.70, 6.50) | 5.70 (4.80, 6.70) | <0.001 |
| UACR, mg/g | 6.40 (4.10, 12.10) | 5.40 (3.70, 9.10) | 5.90 (4.00, 10.67) | 6.70 (4.30, 12.38) | 8.10 (4.80, 19.20) | <0.001 |
| eGFR, mL/min/1.73 m² | 98.10 (82.60, 112.00) | 102.60 (88.20, 116.20) | 97.80 (82.50, 112.57) | 96.25 (81.20, 109.30) | 95.90 (77.90, 109.20) | <0.001 |
| Antihypertensive drugs, n (%) | 2206 (26.5) | 286 (13.8) | 508 (24.4) | 626 (30.1) | 786 (37.8) | <0.001 |
| Antihyperglycemic drugs, n (%) | 674 (8.1) | 58 (2.8) | 117 (5.6) | 182 (8.8) | 317 (15.2) | <0.001 |
| Statin use, n (%) | 1537 (18.5) | 232 (11.2) | 368 (17.7) | 428 (20.6) | 509 (24.5) | <0.001 |
| Hypertension, n (%) | 4202 (50.5) | 677 (32.6) | 1047 (50.4) | 1184 (57.0) | 1294 (62.2) | <0.001 |
| Diabetes, n (%) | 1122 (13.5) | 95 (4.6) | 185 (8.9) | 279 (13.4) | 563 (27.1) | <0.001 |
| MetS, n (%) | 3089 (37.2) | 171 (8.2) | 565 (27.2) | 910 (43.8) | 1443 (69.4) | <0.001 |
| Liver disease, n (%) | 288 (3.5) | 45 (2.2) | 67 (3.2) | 76 (3.7) | 100 (4.8) | <0.001 |
| Cancer, n (%) | 744 (8.9) | 136 (6.5) | 168 (8.1) | 199 (9.6) | 241 (11.6) | <0.001 |
| CKD, n (%) |  |  |  |  |  | <0.001 |
| Low-risk | 7090 (85.3) | 1899 (91.3) | 1820 (87.6) | 1743 (83.9) | 1628 (78.3) |  |
| Moderate to high-risk | 1120 (13.5) | 171 (8.2) | 237 (11.4) | 310 (14.9) | 402 (19.3) |  |
| Very high-risk | 104 (1.3) | 9 (0.4) | 21 (1.0) | 25 (1.2) | 49 (2.4) |  |
| CKM syndrome, n (%) |  |  |  |  |  | <0.001 |
| Stage 0 | 856 (10.3) | 580 (27.9) | 178 (8.6) | 80 (3.8) | 18 (0.9) |  |
| Stage 1 | 1660 (20.0) | 625 (30.1) | 519 (25.0) | 357 (17.2) | 159 (7.6) |  |
| Stage 2 | 4427 (53.2) | 724 (34.8) | 1050 (50.5) | 1271 (61.2) | 1382 (66.5) |  |
| Stage 3 | 584 (7.0) | 61 (2.9) | 131 (6.3) | 157 (7.6) | 235 (11.3) |  |
| Stage 4 | 787 (9.5) | 89 (4.3) | 200 (9.6) | 213 (10.3) | 285 (13.7) |  |
| Advanced CKM stages, n (%) | 1371 (16.5) | 150 (7.2) | 331 (15.9) | 370 (17.8) | 520 (25.0) | <0.001 |

Abbreviations: CTI, C-reactive protein-triglyceride glucose index; PIR, ratio of family income to poverty; BMI, body mass index; SBP, systolic blood pressure; DBP, diastolic blood pressure; FBG, fasting blood glucose; HbA1c, hemoglobin A1c; TG, triglyceride; TC, total cholesterol; HDL, high density lipoprotein cholesterol; LDL, low density lipoprotein cholesterol; CRP, C-reactive protein; UA, uric acid; UACR, urine albumin-to-creatinine ratio; eGFR, estimated glomerular filtration rate; MeTS, metabolic syndrome; CKD, chronic kidney disease; CKM, Cardiovascular-Kidney-Metabolic

Normality was assessed using the Kolmogorov-Smirnov test, and all continuous variables were non-normally distributed, thus described as median (interquartile range); categorical variables were expressed as frequency (percentage)

**Table S9. Discrimination and reclassification statistics (95% CI) for all-cause and cardiovascular mortality after addition of CTI to the reference clinical covariate model**

|  | **C statistic** | | **NRI** | **IDI** |
| --- | --- | --- | --- | --- |
| **Model** | **Estimates** | **Difference** |  |  |
| **All-cause mortality** |  |  |  |  |
| Reference model | 0.865 (0.857-0.873) | Ref | Ref | Ref |
| + CTI | 0.867 (0.858-0.875) | 0.2% (0.1%-0.3%) | 0.175 (0.091-0.260) | 0.004 (0.001-0.008) |
| **CVD mortality** |  |  |  |  |
| Reference model | 0.884 (0.871-0.897) | Ref | Ref | Ref |
| + CTI | 0.887 (0.874-0.900) | 0.2% (0.1%-0.5%) | 0.280 (0.130-0.405) | 0.004 (0.001-0.012) |

The reference model included Age, Gender, Race, Marital, Education, Smoking, Drinking, PIR, BMI, SBP, HDL, LDL, HbA1c, UA, eGFR, UACR, Statin use, Antihypertensive drugs, Antihyperglycemic drugs, Liver disease, Cancer.

Differences in the C-statistic were estimated as the absolute increase after adding CTI to the reference model. The 95% CIs for differences were estimated using 1000 bootstrap resamples. The C statistic represents the overall concordance index from Cox models, whereas NRI and IDI were calculated for 10-year mortality risk.

Abbreviations: CTI, C-reactive protein-triglyceride glucose index; CVD, cardiovascular disease; NRI, net reclassification improvement; IDI, integrated discrimination improvement; CI, confidence interval; PIR, ratio of family income to poverty; BMI, body mass index; SBP, systolic blood pressure; HDL, high density lipoprotein cholesterol; LDL, low density lipoprotein cholesterol; HbA1c, hemoglobin A1c; UA, uric acid; eGFR, estimated glomerular filtration rate; UACR, urine albumin-to-creatinine ratio

|  | **Model 1**  **OR (95%CI)** | ***P*-value** | **Model 2**  **OR (95%CI)** | ***P*-value** | **Model 3**  **OR (95%CI)** | ***P*-value** | **Model 4**  **OR (95%CI)** | ***P*-value** |
| --- | --- | --- | --- | --- | --- | --- | --- | --- |
| Per SD increase | 1.76 (1.64, 1.88) | <0.001 | 1.67 (1.52, 1.84) | <0.001 | 1.57 (1.43, 1.74) | <0.001 | 1.22 (1.06, 1.41) | 0.008 |
| Q1 | Ref |  | Ref |  | Ref |  | Ref |  |
| Q2 | 2.69 (2.12, 3.41) | <0.001 | 1.95 (1.44, 2.63) | <0.001 | 1.83 (1.35, 2.47) | <0.001 | 1.51 (1.10, 2.08) | 0.013 |
| Q3 | 3.03 (2.42, 3.80) | <0.001 | 1.80 (1.36, 2.38) | <0.001 | 1.62 (1.22, 2.14) | <0.001 | 1.19 (0.87, 1.62) | 0.273 |
| Q4 | 5.24 (4.19, 6.55) | <0.001 | 3.74 (2.87, 4.86) | <0.001 | 3.21 (2.45, 4.22) | <0.001 | 1.92 (1.33, 2.76) | <0.001 |
| *P* for trend |  | <0.001 |  | <0.001 |  | <0.001 |  | 0.005 |

**Table S10. Association between CTI and advanced cardiovascular-kidney-metabolic stages excluding events occurred in the first two year of follow up**

SD of CTI = 0.66.

Model 1: unadjusted; Model 2: adjusted for Age, Gender, Race; Model 3: adjusted for Age, Gender, Race, Marital, Education, Smoking and Drinking; Model 4: adjusted for Age, Gender, Race, Marital, Education, Smoking, Drinking, PIR, BMI, SBP, HDL, LDL, HbA1c, UA, eGFR, UACR, Statin use, Antihypertensive drugs, Antihyperglycemic drugs, Liver disease, Cancer

Abbreviations: CTI, C-reactive protein-triglyceride glucose index; CI, confidence interval; OR, odds ratio; PIR, ratio of family income to poverty; BMI, body mass index; SBP, systolic blood pressure; HDL, high density lipoprotein cholesterol; LDL, low density lipoprotein cholesterol; HbA1c, hemoglobin A1c; UA, uric acid; eGFR, estimated glomerular filtration rate; UACR, urine albumin-to-creatinine ratio

**Table S11. Association between CTI and All-Cause and cardiovascular Mortality stratified by Advanced CKM stages excluding events occurred in the first two year of follow up**

|  |  | **All participants** |  | **Non-Advanced CKM stages** | | | **Advanced CKM stages** | | |  |
| --- | --- | --- | --- | --- | --- | --- | --- | --- | --- | --- |
|  | **Events/Total (%)** | **HR & 95%CI** | ***P*-value** | **Events/Total (%)** | **HR & 95%CI** | ***P*-value** | **Events/Total (%)** | **HR & 95%CI** | ***P*-value** | ***P*-interaction** |
| **All-cause mortality** | | | | | | | | | | |
| Per SD increase | 1468/8176 (17.9) | 1.31 (1.20, 1.44) | <0.001 | 748/6892 (10.8) | 1.32 (1.15, 1.51) | <0.001 | 720/1284 (56.0) | 1.32 (1.17, 1.50) | <0.001 | 0.150 |
| Q1 | 181/2044 (8.8) | Ref | - | 105/1902 (5.5) | Ref | - | 76/142 (53.5) | Ref | - | 0.547 |
| Q2 | 336/2044 (16.4) | 1.20 (0.97, 1.49) | 0.093 | 169/1728 (9.7) | 1.25 (0.95, 1.66) | 0.117 | 167/316 (52.8) | 1.12 (0.81, 1.57) | 0.489 |  |
| Q3 | 433/2044 (21.1) | 1.33 (1.07, 1.67) | 0.012 | 237/1700 (13.9) | 1.33 (0.98, 1.79) | 0.065 | 196/344 (56.9) | 1.39 (1.01, 1.92) | 0.046 |  |
| Q4 | 518/2044 (25.3) | 1.69 (1.34, 2.13) | <0.001 | 237/1562 (15.1) | 1.67 (1.18, 2.37) | 0.004 | 281/482 (58.2) | 1.69 (1.23, 2.32) | 0.001 |  |
| *P* for trend | - | - | <0.001 | - | - | 0.006 | - | - | <0.001 | - |
| **CVD mortality** | | | | | | | | | | |
| Per SD increase | 433/8176 (5.2) | 1.37 (1.17, 1.60) | <0.001 | 193/6892 (2.8) | 1.45 (1.15, 1.83) | <0.001 | 240/1284 (18.6) | 1.29 (1.05, 1.59) | 0.018 | 0.322 |
| Q1 | 45/2044 (2.2) | Ref | - | 24/1902 (1.2) | Ref | - | 21/142 (14.7) | Ref | - | 0.659 |
| Q2 | 110/2044 (5.3) | 1.73 (1.10, 2.72) | 0.017 | 41/1728 (2.3) | 1.74 (0.98, 3.08) | 0.059 | 69/316 (21.8) | 1.55 (0.81, 2.95) | 0.184 |  |
| Q3 | 122/2044 (5.9) | 1.56 (1.01, 2.43) | 0.045 | 66/1700 (3.8) | 1.87 (1.00, 3.51) | 0.051 | 56/344 (16.2) | 1.25 (0.68, 2.32) | 0.474 |  |
| Q4 | 156/2044 (7.6) | 2.31 (1.48, 3.60) | <0.001 | 62/1562 (3.9) | 2.49 (1.30, 4.76) | 0.006 | 94/482 (19.5) | 1.94 (1.07, 3.51) | 0.029 |  |
| *P* for trend | - | - | <0.001 | - | - | 0.009 | - | - | 0.055 | - |

Full adjustment for Age, Gender, Race, Marital, Education, Smoking, Drinking, PIR, BMI, SBP, HDL, LDL, HbA1c, UA, eGFR, UACR, Statin use, Antihypertensive drugs, Antihyperglycemic drugs, Liver disease, Cancer

The denominator for each CKM subgroup remained unchanged across both the all-cause and cardiovascular mortality analyses; cardiovascular deaths represent a subset of all-cause deaths within the same analytic sample.

Abbreviations: CTI, C-reactive protein-triglyceride glucose index; CKM, cardiovascular-kidney-metabolic; CI, confidence interval; HR, hazard ratio; PIR, ratio of family income to poverty; BMI, body mass index; SBP, systolic blood pressure; HDL, high density lipoprotein cholesterol; LDL, low density lipoprotein cholesterol; HbA1c, hemoglobin A1c; UA, uric acid; eGFR, estimated glomerular filtration rate; UACR, urine albumin-to-creatinine ratio

**Table S12.Association between CTI and advanced cardiovascular-kidney-metabolic stages excluding individuals with a history of cancer**

|  | **Model 1**  **OR (95%CI)** | ***P*-value** | **Model 2**  **OR (95%CI)** | ***P*-value** | **Model 3**  **OR (95%CI)** | ***P*-value** | **Model 4**  **OR (95%CI)** | ***P*-value** |
| --- | --- | --- | --- | --- | --- | --- | --- | --- |
| Per SD increase | 1.83 (1.70, 1.97) | <0.001 | 1.70 (1.52, 1.89) | <0.001 | 1.60 (1.42, 1.79) | <0.001 | 1.27 (1.08, 1.49) | 0.005 |
| Q1 | Ref |  | Ref |  | Ref |  | Ref |  |
| Q2 | 2.45 (1.82, 3.28) | <0.001 | 1.66 (1.16, 2.38) | 0.007 | 1.56 (1.08, 2.24) | 0.019 | 1.30 (0.88, 1.90) | 0.191 |
| Q3 | 2.97 (2.30, 3.82) | <0.001 | 1.67 (1.23, 2.27) | 0.001 | 1.49 (1.09, 2.03) | 0.015 | 1.12 (0.80, 1.56) | 0.517 |
| Q4 | 5.39 (4.21, 6.89) | <0.001 | 3.56 (2.60, 4.87) | <0.001 | 3.04 (2.19, 4.22) | <0.001 | 1.92 (1.26, 2.93) | 0.004 |
| *P* for trend |  | <0.001 |  | <0.001 |  | <0.001 |  | 0.004 |

SD of CTI = 0.66.

Model 1: unadjusted; Model 2: adjusted for Age, Gender, Race; Model 3: adjusted for Age, Gender, Race, Marital, Education, Smoking and Drinking; Model 4: adjusted for Age, Gender, Race, Marital, Education, Smoking, Drinking, PIR, BMI, SBP, HDL, LDL, HbA1c, UA, eGFR, UACR, Statin use, Antihypertensive drugs, Antihyperglycemic drugs, Liver disease

Abbreviations: CTI, C-reactive protein-triglyceride glucose index; CI, confidence interval; OR, odds ratio; PIR, ratio of family income to poverty; BMI, body mass index; SBP, systolic blood pressure; HDL, high density lipoprotein cholesterol; LDL, low density lipoprotein cholesterol; HbA1c, hemoglobin A1c; UA, uric acid; eGFR, estimated glomerular filtration rate; UACR, urine albumin-to-creatinine ratio

**Table S13.Association between CTI and All-Cause and cardiovascular Mortality stratified by Advanced CKM stages excluding individuals with a history of cancer**

|  |  | **All participants** |  | **Non-Advanced CKM stages** | | | **Advanced CKM stages** | | |  |
| --- | --- | --- | --- | --- | --- | --- | --- | --- | --- | --- |
|  | **Events/Total (%)** | **HR & 95%CI** | ***P*-value** | **Events/Total (%)** | **HR & 95%CI** | ***P*-value** | **Events/Total (%)** | **HR & 95%CI** | ***P*-value** | ***P*-interaction** |
| **All-cause mortality** | | | | | | | | | | |
| Per SD increase | 1278/7570 (16.8) | 1.34 (1.20, 1.49) | <0.001 | 676/6491 (10.4) | 1.33 (1.14, 1.55) | <0.001 | 602/1079 (55.7) | 1.37 (1.20, 1.56) | <0.001 | 0.186 |
| Q1 | 153/1893 (8.0) | Ref | - | 89/1774 (5.0) | Ref | - | 64/119 (53.7) | Ref | - | 0.387 |
| Q2 | 287/1892 (15.1) | 1.19 (0.94, 1.51) | 0.146 | 157/1641 (9.5) | 1.28 (0.91, 1.78) | 0.155 | 130/251 (51.7) | 1.00 (0.74, 1.34) | 0.987 |  |
| Q3 | 370/1892 (19.5) | 1.30 (1.02, 1.66) | 0.035 | 206/1604 (12.8) | 1.29 (0.92, 1.82) | 0.140 | 164/288 (56.9) | 1.27 (0.93, 1.75) | 0.138 |  |
| Q4 | 468/1893 (24.7) | 1.76 (1.34, 2.31) | <0.001 | 224/1472 (15.2) | 1.75 (1.16, 2.64) | 0.007 | 244/421 (57.9) | 1.60 (1.16, 2.22) | 0.004 |  |
| *P* for trend | - | - | <0.001 | - | - | 0.013 | - | - | 0.002 | - |
| **CVD mortality** | | | | | | | | | | |
| Per SD increase | 399/7570 (5.2) | 1.41 (1.19, 1.68) | <0.001 | 185/6491 (2.9) | 1.47 (1.16, 1.87) | 0.001 | 214/1079 (19.8) | 1.33 (1.04, 1.70) | 0.025 | 0.175 |
| Q1 | 43/1893 (2.2) | Ref | - | 22/1774 (1.2) | Ref | - | 21/119 (17.6) | Ref | - | 0.409 |
| Q2 | 96/1892 (5.0) | 1.73 (1.12, 2.69) | 0.014 | 40/1641 (2.4) | 1.89 (1.05, 3.40) | 0.034 | 56/251 (22.3) | 1.38 (0.76, 2.52) | 0.290 |  |
| Q3 | 112/1892 (5.9) | 1.56 (0.95, 2.55) | 0.076 | 61/1604 (3.8) | 1.86 (0.91, 3.81) | 0.090 | 51/288 (17.7) | 1.14 (0.63, 2.06) | 0.669 |  |
| Q4 | 148/1893 (7.8) | 2.30 (1.44, 3.69) | <0.001 | 62/1472 (4.2) | 2.59 (1.30, 5.18) | 0.007 | 86/421 (20.4) | 1.72 (0.93, 3.18) | 0.086 |  |
| *P* for trend | - | - | 0.004 | - | - | 0.023 | - | - | 0.167 | - |

Full adjustment for Age, Gender, Race, Marital, Education, Smoking, Drinking, PIR, BMI, SBP, HDL, LDL, HbA1c, UA, eGFR, UACR, Statin use, Antihypertensive drugs, Antihyperglycemic drugs, Liver disease

The denominator for each CKM subgroup remained unchanged across both the all-cause and cardiovascular mortality analyses; cardiovascular deaths represent a subset of all-cause deaths within the same analytic sample.

Abbreviations: CTI, C-reactive protein-triglyceride glucose index; CKM, cardiovascular-kidney-metabolic; CI, confidence interval; HR, hazard ratio; PIR, ratio of family income to poverty; BMI, body mass index; SBP, systolic blood pressure; HDL, high density lipoprotein cholesterol; LDL, low density lipoprotein cholesterol; HbA1c, hemoglobin A1c; UA, uric acid; eGFR, estimated glomerular filtration rate; UACR, urine albumin-to-creatinine ratio

**Table S14.Association between CTI and advanced cardiovascular-kidney-metabolic stages excluding those with any missing values**

|  | **Model 1**  **OR (95%CI)** | ***P*-value** | **Model 2**  **OR (95%CI)** | ***P*-value** | **Model 3**  **OR (95%CI)** | ***P*-value** | **Model 4**  **OR (95%CI)** | ***P*-value** |
| --- | --- | --- | --- | --- | --- | --- | --- | --- |
| Per SD increase | 1.79 (1.67, 1.93) | <0.001 | 1.67 (1.50, 1.86) | <0.001 | 1.58 (1.42, 1.75) | <0.001 | 1.25 (1.07, 1.47) | 0.006 |
| Q1 | Ref |  | Ref |  | Ref |  | Ref |  |
| Q2 | 2.51 (1.93, 3.25) | <0.001 | 1.74 (1.26, 2.39) | 0.001 | 1.66 (1.20, 2.28) | 0.003 | 1.36 (0.96, 1.93) | 0.091 |
| Q3 | 3.14 (2.52, 3.90) | <0.001 | 1.80 (1.39, 2.34) | <0.001 | 1.63 (1.25, 2.12) | <0.001 | 1.16 (0.86, 1.58) | 0.338 |
| Q4 | 5.13 (3.99, 6.59) | <0.001 | 3.44 (2.54, 4.65) | <0.001 | 2.99 (2.19, 4.07) | <0.001 | 1.76 (1.18, 2.63) | 0.008 |
| *P* for trend |  | <0.001 |  | <0.001 |  | <0.001 |  | 0.016 |

SD of CTI = 0.64.

Model 1: unadjusted; Model 2: adjusted for Age, Gender, Race; Model 3: adjusted for Age, Gender, Race, Marital, Education, Smoking and Drinking; Model 4: adjusted for Age, Gender, Race, Marital, Education, Smoking, Drinking, PIR, BMI, SBP, HDL, LDL, HbA1c, UA, eGFR, UACR, Statin use, Antihypertensive drugs, Antihyperglycemic drugs, Liver disease, Cancer

Abbreviations: CTI, C-reactive protein-triglyceride glucose index; CI, confidence interval; OR, odds ratio; PIR, ratio of family income to poverty; BMI, body mass index; SBP, systolic blood pressure; HDL, high density lipoprotein cholesterol; LDL, low density lipoprotein cholesterol; HbA1c, hemoglobin A1c; UA, uric acid; eGFR, estimated glomerular filtration rate; UACR, urine albumin-to-creatinine ratio

**Table S15.Association between CTI and All-Cause and cardiovascular Mortality stratified by Advanced CKM stages excluding those with any missing values**

|  |  | **All participants** |  | **Non-Advanced CKM stages** | | | **Advanced CKM stages** | | |  |
| --- | --- | --- | --- | --- | --- | --- | --- | --- | --- | --- |
|  | **Events/Total (%)** | **HR & 95%CI** | ***P*-value** | **Events/Total (%)** | **HR & 95%CI** | ***P*-value** | **Events/Total (%)** | **HR & 95%CI** | ***P*-value** | ***P*-interaction** |
| **All-cause mortality** | | | | | | | | | | |
| Per SD increase | 1294/6887 (18.7) | 1.36 (1.24, 1.50) | <0.001 | 642/5757 (11.1) | 1.37 (1.19, 1.57) | <0.001 | 652/1130 (57.6) | 1.36 (1.20, 1.56) | <0.001 | 0.254 |
| Q1 | 157/1722 (9.1) | Ref | - | 87/1594 (5.4) | Ref | - | 70/128 (54.6) | Ref | - | 0.356 |
| Q2 | 295/1722 (17.1) | 1.29 (1.03, 1.61) | 0.024 | 151/1453 (10.3) | 1.41 (1.04, 1.91) | 0.027 | 144/269 (53.5) | 1.12 (0.84, 1.50) | 0.425 |  |
| Q3 | 373/1721 (21.6) | 1.33 (1.04, 1.70) | 0.025 | 192/1413 (13.5) | 1.33 (0.95, 1.88) | 0.099 | 181/308 (58.7) | 1.37 (1.01, 1.87) | 0.047 |  |
| Q4 | 469/1722 (27.2) | 1.84 (1.43, 2.36) | <0.001 | 212/1297 (16.3) | 1.92 (1.32, 2.80) | <0.001 | 257/425 (60.4) | 1.73 (1.26, 2.39) | <0.001 |  |
| *P* for trend | - | - | <0.001 | - | - | 0.002 | - | - | <0.001 | - |
| **CVD mortality** | | | | | | | | | | |
| Per SD increase | 388/6887 (5.6) | 1.47 (1.23, 1.76) | <0.001 | 166/5757 (2.8) | 1.58 (1.24, 2.02) | <0.001 | 222/1130 (19.6) | 1.29 (1.03, 1.62) | 0.025 | 0.066 |
| Q1 | 41/1722 (2.3) | Ref | - | 19/1594 (1.1) | Ref | - | 22/128 (17.1) | Ref | - | 0.187 |
| Q2 | 94/1722 (5.4) | 1.74 (1.10, 2.75) | 0.019 | 33/1453 (2.2) | 1.87 (1.01, 3.48) | 0.048 | 61/269 (22.6) | 1.37 (0.77, 2.43) | 0.283 |  |
| Q3 | 108/1721 (6.2) | 1.65 (1.06, 2.57) | 0.027 | 57/1413 (4.0) | 2.12 (1.14, 3.92) | 0.017 | 51/308 (16.5) | 1.11 (0.61, 2.03) | 0.725 |  |
| Q4 | 145/1722 (8.4) | 2.59 (1.64, 4.07) | <0.001 | 57/1297 (4.3) | 3.03 (1.71, 5.36) | <0.001 | 88/425 (20.7) | 1.86 (1.01, 3.41) | 0.047 |  |
| *P* for trend | - | - | 0.001 | - | - | 0.001 | - | - | 0.057 | - |

Full adjustment for Age, Gender, Race, Marital, Education, Smoking, Drinking, PIR, BMI, SBP, HDL, LDL, HbA1c, UA, eGFR, UACR, Statin use, Antihypertensive drugs, Antihyperglycemic drugs, Liver disease, Cancer

The denominator for each CKM subgroup remained unchanged across both the all-cause and cardiovascular mortality analyses; cardiovascular deaths represent a subset of all-cause deaths within the same analytic sample.

Abbreviations: CTI, C-reactive protein-triglyceride glucose index; CKM, cardiovascular-kidney-metabolic; CI, confidence interval; HR, hazard ratio; PIR, ratio of family income to poverty; BMI, body mass index; SBP, systolic blood pressure; HDL, high density lipoprotein cholesterol; LDL, low density lipoprotein cholesterol; HbA1c, hemoglobin A1c; UA, uric acid; eGFR, estimated glomerular filtration rate; UACR, urine albumin-to-creatinine ratio

**Table S16. Association between CTI and All-Cause and cardiovascular Mortality stratified by CKM stages (0-2, 3, 4)**

|  | **Non-Advanced CKM stages** | | | **CKM Stage 3** | | | **CKM Stage 4** | | | ***P*-interaction** |
| --- | --- | --- | --- | --- | --- | --- | --- | --- | --- | --- |
|  | **Events/Total (%)** | **HR & 95%CI** | ***P*-value** | **Events/Total (%)** | **HR & 95%CI** | ***P*-value** | **Events/Total (%)** | **HR & 95%CI** | ***P*-value** |  |
| **All-cause mortality** | | | | | | | | | | |
| Per SD increase | 799/6943 (11.5) | 1.35 (1.19, 1.54) | <0.001 | 393/584 (67.2) | 1.35 (1.14, 1.60) | <0.001 | 414/787 (52.6) | 1.51 (1.30, 1.75) | <0.001 | 0.062 |
| Q1 | 110/1929 (5.7) | Ref | - | 41/61 (67.2) | Ref | - | 43/89 (48.3) | Ref | - | 0.090 |
| Q2 | 183/1747 (10.4) | 1.31 (0.99, 1.73) | 0.062 | 90/131 (68.7) | 0.85 (0.57, 1.28) | 0.431 | 91/200 (45.5) | 1.34 (0.87, 2.09) | 0.186 |  |
| Q3 | 246/1708 (14.4) | 1.36 (1.02, 1.82) | 0.038 | 110/157 (70.0) | 1.26 (0.83, 1.93) | 0.277 | 110/213 (51.6) | 1.47 (0.99, 2.18) | 0.058 |  |
| Q4 | 260/1559 (16.6) | 1.84 (1.32, 2.56) | <0.001 | 152/235 (64.6) | 1.32 (0.85, 2.05) | 0.217 | 170/285 (59.6) | 2.41 (1.60, 3.64) | <0.001 |  |
| *P* for trend | - | - | <0.001 | - | - | 0.046 | - | - | <0.001 | - |
| **CVD mortality** | | | | | | | | | | |
| Per SD increase | 204/6943 (2.9) | 1.48 (1.20, 1.84) | <0.001 | 112/584 (19.2) | 1.72 (1.24, 2.40) | 0.001 | 159/787 (20.2) | 1.23 (0.92, 1.65) | 0.155 | 0.632 |
| Q1 | 25/1929 (1.2) | Ref | - | 11/61 (18.0) | Ref | - | 17/89 (19.1) | Ref | - | 0.805 |
| Q2 | 44/1747 (2.5) | 1.85 (1.08, 3.15) | 0.024 | 29/131 (22.1) | 1.17 (0.55, 2.48) | 0.682 | 41/200 (20.5) | 1.19 (0.54, 2.61) | 0.662 |  |
| Q3 | 69/1708 (4.0) | 1.96 (1.08, 3.53) | 0.026 | 26/157 (16.5) | 1.38 (0.62, 3.04) | 0.429 | 38/213 (17.8) | 0.94 (0.47, 1.89) | 0.862 |  |
| Q4 | 66/1559 (4.2) | 2.75 (1.57, 4.83) | <0.001 | 46/235 (19.5) | 2.34 (1.09, 5.04) | 0.029 | 63/285 (22.1) | 1.45 (0.67, 3.15) | 0.342 |  |
| *P* for trend | - | - | 0.001 | - | - | 0.006 | - | - | 0.392 | - |

Full adjustment for Age, Gender, Race, Marital, Education, Smoking, Drinking, PIR, BMI, SBP, HDL, LDL, HbA1c, UA, eGFR, UACR, Statin use, Antihypertensive drugs, Antihyperglycemic drugs, Liver disease, Cancer

HRs for quartiles (Q2–Q4) were estimated by modeling CTI quartiles as categorical variables, using Q1 as the reference group. The P for trend was calculated by treating CTI quartiles as an ordinal variable (coded 1-4) in the fully adjusted Cox model.

The denominator for each CKM subgroup remained unchanged across both the all-cause and cardiovascular mortality analyses; cardiovascular deaths represent a subset of all-cause deaths within the same analytic sample.

Abbreviations: CTI, C-reactive protein-triglyceride glucose index; CKM, cardiovascular-kidney-metabolic; CI, confidence interval; HR, hazard ratio; PIR, ratio of family income to poverty; BMI, body mass index; SBP, systolic blood pressure; HDL, high density lipoprotein cholesterol; LDL, low density lipoprotein cholesterol; HbA1c, hemoglobin A1c; UA, uric acid; eGFR, estimated glomerular filtration rate; UACR, urine albumin-to-creatinine ratio

**Table S17. Association between CTI and cardiovascular-kidney-metabolic stages (0-4)**

|  | **Model 1**  **OR (95%CI)** | ***P*-value** | **Model 2**  **OR (95%CI)** | ***P*-value** | **Model 3**  **OR (95%CI)** | ***P*-value** | **Model 4**  **OR (95%CI)** | ***P*-value** |
| --- | --- | --- | --- | --- | --- | --- | --- | --- |
| Per SD increase | 2.54 (2.42, 2.67) | <0.001 | 2.35 (2.22, 2.48) | <0.001 | 2.32 (2.19, 2.46) | <0.001 | 1.59 (1.48, 1.71) | <0.001 |
| Q1 | Ref |  | Ref |  | Ref |  | Ref |  |
| Q2 | 3.19 (2.76, 3.69) | <0.001 | 2.68 (2.27, 3.16) | <0.001 | 2.63 (2.23, 3.10) | <0.001 | 1.78 (1.51, 2.09) | <0.001 |
| Q3 | 5.45 (4.76, 6.24) | <0.001 | 4.39 (3.73, 5.17) | <0.001 | 4.29 (3.64, 5.05) | <0.001 | 2.22 (1.87, 2.63) | <0.001 |
| Q4 | 11.21 (9.80, 12.82) | <0.001 | 9.20 (7.82, 10.82) | <0.001 | 8.89 (7.53, 10.50) | <0.001 | 3.32 (2.68, 4.11) | <0.001 |
| *P* for trend |  | <0.001 |  | <0.001 |  | <0.001 |  | <0.001 |

SD of CTI = 0.66.

Model 1: unadjusted; Model 2: adjusted for Age, Gender, Race; Model 3: adjusted for Age, Gender, Race, Marital, Education, Smoking and Drinking; Model 4: adjusted for Age, Gender, Race, Marital, Education, Smoking, Drinking, PIR, BMI, SBP, HDL, LDL, HbA1c, UA, eGFR, Statin use, Antihypertensive drugs, Antihyperglycemic drugs, Liver disease, Cancer

In the fully adjusted model (Model 4), UACR was not included because inclusion of UACR resulted in model non-convergence.

Abbreviations: CTI, C-reactive protein-triglyceride glucose index; CI, confidence interval; OR, odds ratio; PIR, ratio of family income to poverty; BMI, body mass index; SBP, systolic blood pressure; HDL, high density lipoprotein cholesterol; LDL, low density lipoprotein cholesterol; HbA1c, hemoglobin A1c; UA, uric acid; eGFR, estimated glomerular filtration rate

**Table S18. Association between CTI and All-Cause and cardiovascular Mortality using a parsimonious adjustment model**

|  |  | **All participants** |  | **Non-Advanced CKM stages** | | | **Advanced CKM stages** | | |  |
| --- | --- | --- | --- | --- | --- | --- | --- | --- | --- | --- |
|  | **Events/Total (%)** | **HR & 95%CI** | ***P*-value** | **Events/Total (%)** | **HR & 95%CI** | ***P*-value** | **Events/Total (%)** | **HR & 95%CI** | ***P*-value** | ***P*-interaction** |
| **All-cause mortality** | | | | | | | | | | |
| Per SD increase | 1606/8314 (19.3) | 1.34 (1.25, 1.44) | <0.001 | 799/6943 (11.5) | 1.33 (1.20, 1.48) | <0.001 | 807/1371 (58.8) | 1.31 (1.19, 1.44) | <0.001 | 0.518 |
| Q1 | 194/2079 (9.3) | Ref | - | 110/1929 (5.7) | Ref | - | 84/150 (56.0) | Ref | - | 0.769 |
| Q2 | 364/2078 (17.5) | 1.23 (1.02, 1.48) | 0.034 | 183/1747 (10.4) | 1.29 (0.98, 1.70) | 0.071 | 181/331 (54.6) | 1.09 (0.82, 1.47) | 0.547 |  |
| Q3 | 466/2078 (22.4) | 1.31 (1.08, 1.59) | 0.005 | 246/1708 (14.4) | 1.34 (1.04, 1.73) | 0.023 | 220/370 (59.4) | 1.25 (0.92, 1.70) | 0.160 |  |
| Q4 | 582/2079 (27.9) | 1.83 (1.54, 2.18) | <0.001 | 260/1559 (16.6) | 1.83 (1.41, 2.38) | <0.001 | 322/520 (61.9) | 1.63 (1.24, 2.13) | <0.001 |  |
| *P* for trend | - | - | <0.001 | - | - | <0.001 | - | - | <0.001 | - |
| **CVD mortality** | | | | | | | | | | |
| Per SD increase | 475/8314 (5.7) | 1.48 (1.31, 1.69) | <0.001 | 204/6943 (2.9) | 1.46 (1.22, 1.75) | <0.001 | 271/1371 (19.7) | 1.37 (1.17, 1.62) | <0.001 | 0.503 |
| Q1 | 53/2079 (2.5) | Ref | - | 25/1929 (1.2) | Ref | - | 28/150 (18.6) | Ref | - | 0.562 |
| Q2 | 114/2078 (5.4) | 1.68 (1.17, 2.41) | 0.005 | 44/1747 (2.5) | 1.77 (1.05, 2.98) | 0.031 | 70/331 (21.1) | 1.33 (0.81, 2.19) | 0.266 |  |
| Q3 | 133/2078 (6.4) | 1.60 (1.10, 2.33) | 0.013 | 69/1708 (4.0) | 1.93 (1.15, 3.25) | 0.013 | 64/370 (17.2) | 1.17 (0.68, 2.01) | 0.575 |  |
| Q4 | 175/2079 (8.4) | 2.60 (1.90, 3.55) | <0.001 | 66/1559 (4.2) | 2.68 (1.69, 4.24) | <0.001 | 109/520 (20.9) | 1.91 (1.25, 2.93) | 0.003 |  |
| *P* for trend | - | - | <0.001 | - | - | <0.001 | - | - | 0.003 | - |

Parsimonious adjustment for Age, Gender, Race, Marital, Education, Smoking, Drinking, PIR, Liver disease, Cancer

HRs for quartiles (Q2-Q4) were estimated by modeling CTI quartiles as categorical variables, using Q1 as the reference group. The P for trend was calculated by treating CTI quartiles as an ordinal variable (coded 1-4) in the fully adjusted Cox model.

The denominator for each CKM subgroup remained unchanged across both the all-cause and cardiovascular mortality analyses; cardiovascular deaths represent a subset of all-cause deaths within the same analytic sample.

Abbreviations: CTI, C-reactive protein-triglyceride glucose index; CKM, cardiovascular-kidney-metabolic; CI, confidence interval; HR, hazard ratio; PIR, ratio of family income to poverty;

**
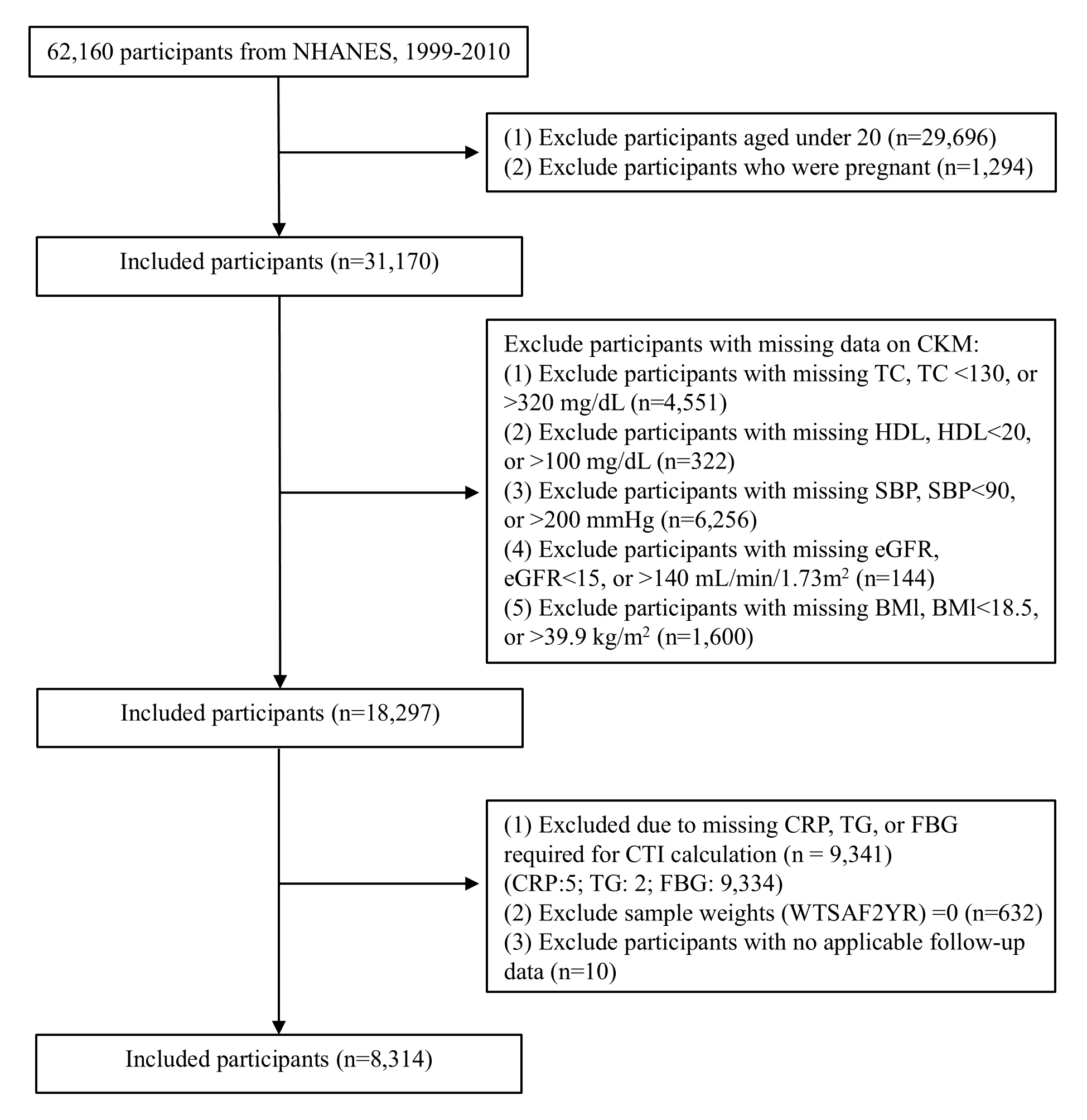
Figure S1. Participant Selection Process Flowchart**

**Figure S2.Restricted cubic splines illustrate the relationship between CTI and advanced CKM stages.**


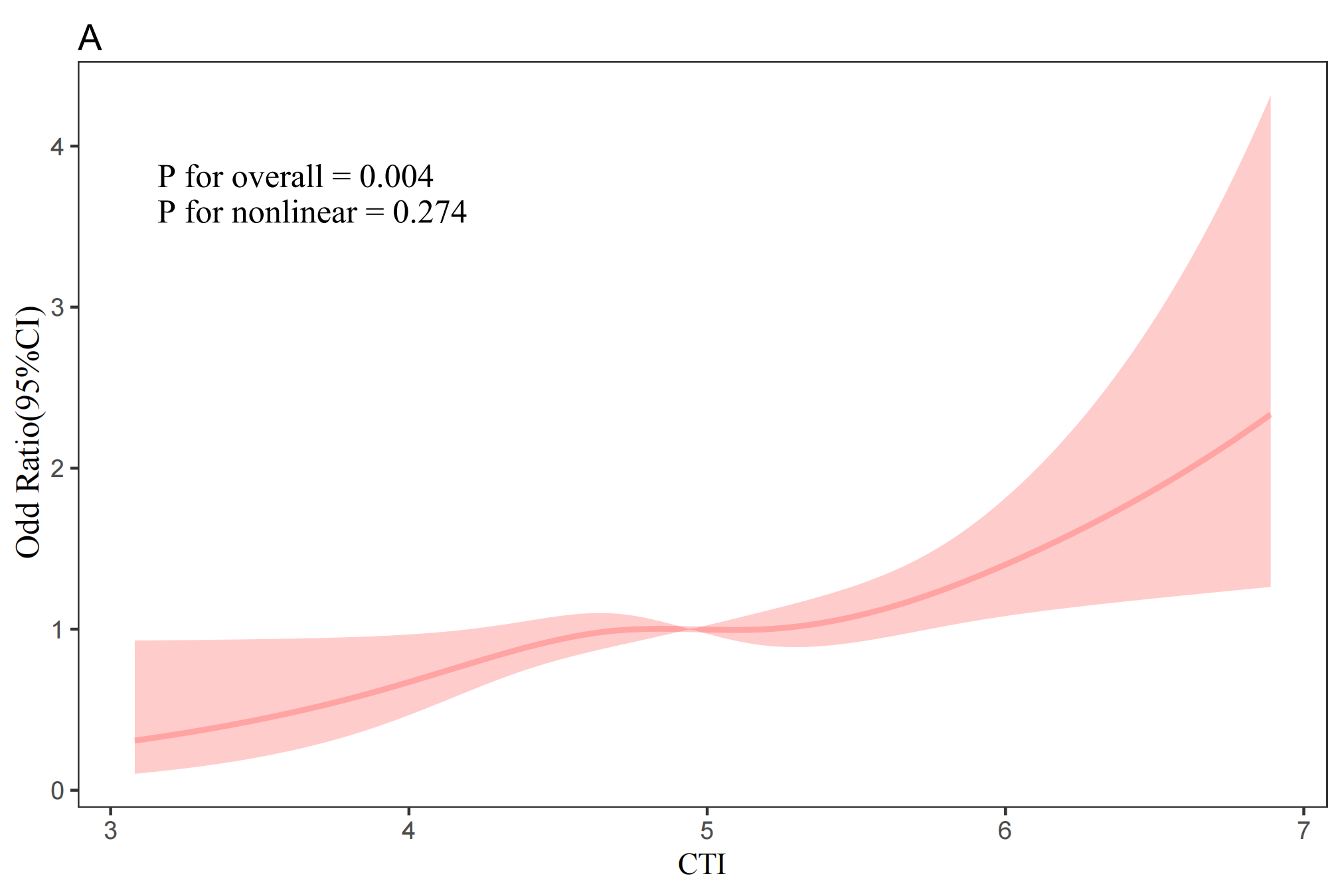


Full adjustment for Age, Gender, Race, Marital, Education, Smoking, Drinking, PIR, BMI, SBP, HDL, LDL, HbA1c, UA, eGFR, UACR, Statin use, Antihypertensive drugs, Antihyperglycemic drugs, Liver disease, Cancer. CTI, C-reactive protein-triglyceride glucose index; CKM, Cardiovascular-Kidney-Metabolic

**Figure S3.Kaplan–Meier analyses for all-cause (A) and cardiovascular mortality (B) across different C-reactive protein-triglyceride glucose index groups**


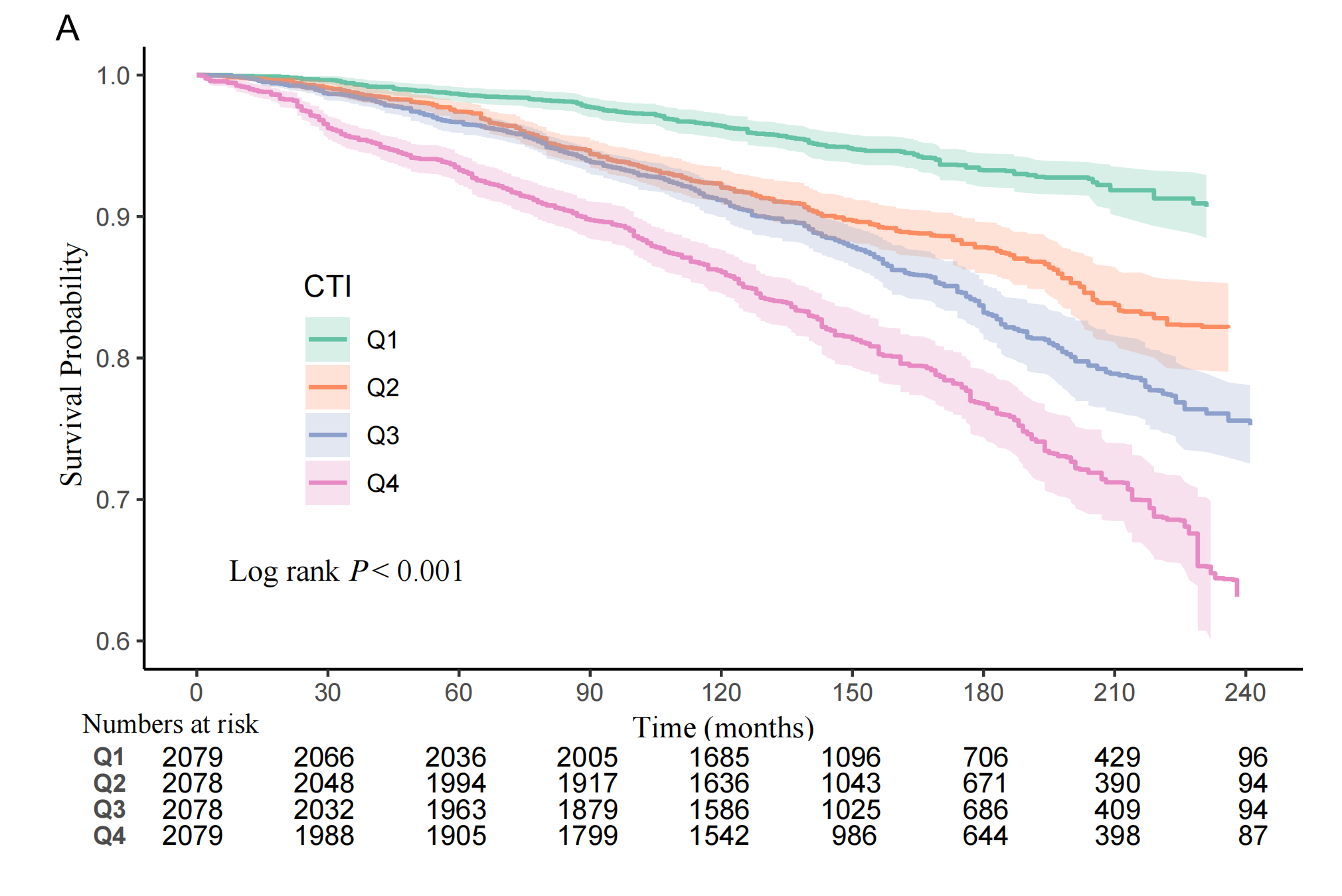

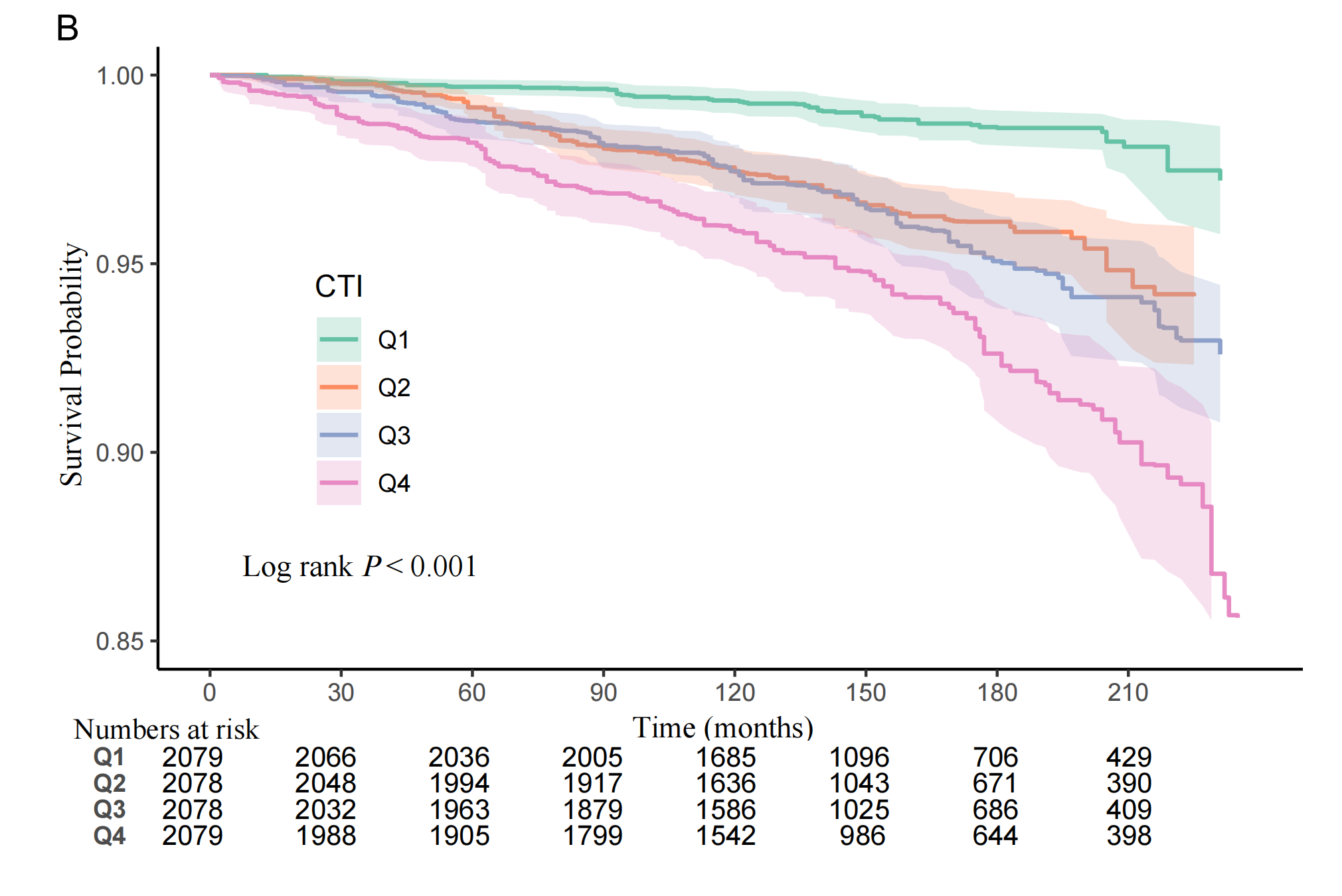


**
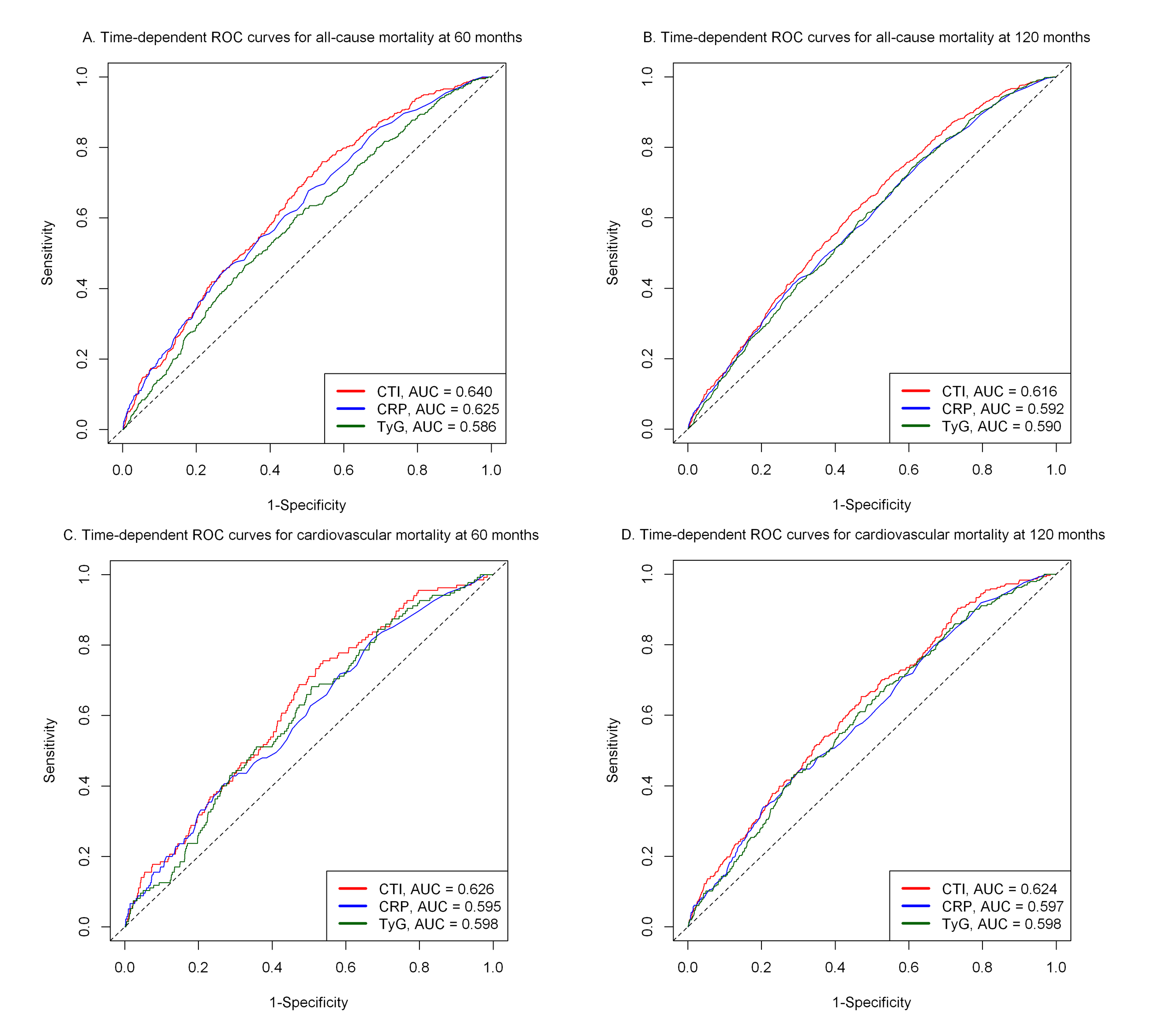
Figure S4. Time-dependent receiver operating characteristic (ROC) curves for all-cause mortality and cardiovascular mortality at 60 and 120 months**
